# Influence of taping on force sense accuracy: a systematic review with between and within group meta-analysis

**DOI:** 10.1186/s13102-023-00740-1

**Published:** 2023-10-20

**Authors:** Shashank Ghai, Ishan Ghai, Susanne Narciss

**Affiliations:** 1 Department of Political, Historical, Religious and Cultural Studies, Karlstad University, Karlstad, Sweden; 2 Centre for Societal Risk Research, Karlstad University, Karlstad, Sweden; 3 Psychology of Learning and Instruction, Department of Psychology, School of Science, Technische Universität Dresden, Dresden, Germany; 4 Centre for Tactile Internet With Human-in-the-Loop (CeTI), Technische Universität Dresden, Dresden, Germany; 5 School of Life Sciences, Jacobs University Bremen, Bremen, Germany

**Keywords:** Taping, Tactile stimulation, Kinetics, Force sense error, Sensorimotor learning

## Abstract

Taping is a common technique used to address proprioceptive deficits in both healthy and patient population groups. Although there is increasing interest in taping to address proprioceptive deficits, little is known about its effects on the kinetic aspects of proprioception as measured by force sense accuracy. To address this gap in the literature, the present systematic review and meta-analysis was conducted to evaluate the impact of taping on force sense accuracy. A search for relevant literature was conducted following PRISMA guidelines across seven databases and one register. Eleven studies with 279 participants were included in the review out of 7362 records. In the between-group analyses, we found a significant improvement in absolute (*p* < 0.01) and relative (*p* = 0.01) force sense accuracy with taping compared to no comparator. Likewise, a significant improvement in absolute (*p* = 0.01) force sense accuracy was also observed with taping compared to placebo tape. In the within group analysis, this reduction in the absolute (*p* = 0.11) force sense accuracy was not significant. Additional exploratory subgroup analyses revealed between group improvement in force sense accuracy in both healthy individuals and individuals affected by medial epicondylitis. The findings of this meta-analysis should be interpreted with caution due to the limited number of studies and a lack of blinded randomized controlled trials, which may impact the generalizability of the results. More high-quality research is needed to confirm the overall effect of taping on force sense accuracy.

**Supplementary Information:**

The online version contains supplementary material available at 10.1186/s13102-023-00740-1.

## Introduction

Taping has gained widespread attention in rehabilitation and performance science [1]. The earliest use of taping in rehabilitation dates back to as early as 1969, as documented in the literature [2]. Its integration in modern practice was prominently observed during the 2012 London Olympics, where Kinesiotaping was identified as one of the most frequently used treatment modalities by the Olympians [3]. The intervention entails the application of specialized adhesive tape to the body to achieve various therapeutic and performance-related outcomes [4]. The growing use of this intervention is largely due to its viability [5], ease of application [6], availability [7], and cost-effectiveness [8, 9]. Owing to these factors, the application of taping in the existing literature extends across a range of medical conditions including chronic ankle instability [10], patellofemoral pain syndrome [11], low back pain [12], lymphedema [13], Parkinson’s disease [14], and stroke [15]. Likewise, the application of taping also extends across a range of sports including, Judo [16], athletics [17], Taekwondo [18], soccer [19], and Karate [20].

Researchers have put forth various explanations concerning the effects of taping in the literature [10, 21–25]. For instance, adhesive patellar taping has been reported to alternate muscle function by facilitating muscle kinematics such as, torque, moment, and power [21]. Similarly, in individuals with patellofemoral pain syndrome [26], the application of adhesive patellar taping has been reported to allocate knee extensor moment arm in an advantageous position which results in facilitated muscle activity. Additionally, facilitating ergonomic movement strategies including trunk engagement, and improvement of vastus medialis obliques and vastus lateralis ratio have been reported with rigid Leuko taping to be supplementary mechanisms that could facilitate muscle function [27]. Likewise, “microcirculatory” effects of Kinesiotaping interventions have also been proposed as a mechanism in the literature that could facilitate recovery. Studies have suggested that the “skin lifting” associated with taping interventions could facilitate local circulation [28], augment lymphatic and venous drainage [29, 30] which might result in enhanced recovery and performance. Moreover, taping has also been reported to pertain with psychological effects, primarily centered around boosting the wearer’s confidence and perception of stability [10, 31, 32]. Researchers have indicated that heightened confidence and a sense of stability through rigid sports taping could enable individuals with joint instability to perform challenging tasks more effectively than without the tape [10, 31].

Furthermore, an additional advantage attributed to taping is the enhancement of joint proprioception. This aspect has been linked to taping’s potential to improve recovery and performance [33–35]. Research has suggested that the tactile feedback provided by taping activates mechanoreceptors, which can enhance proprioception through increased input to the central pathways [34, 36]. Eventually, this amplified afferent input consequently aids in bolstering the efferent neuromuscular response, which increases both the speed and the quality of the muscle reaction (i.e., reduced reaction time) [37, 38]. Konishi [36] suggested that reduced proprioceptive activity due to injury or weakness might impede the ability of mechanoreceptors to provide regular afferent feedback to gamma motor neurons. This deficiency in afferent feedback could disrupt the modulation of primary afferents and hinder the recruitment of high-threshold motor units [39]. The author suggested that taping’s tactile stimulation might offer a potential solution by bypassing this deficit and rescuing alpha motor activity [36].

Additionally, elastic tapes such as Kinesiotape have been reported to induce a phenomenon known as “skin stretch”, which alters underlying tissue and improves proprioception [40, 41]. This ability of the taping to influence underlying tissue is believed to modify the musculoskeletal kinetics, ultimately leading to improved muscular activation [42–44], and increased comfort [45]. For instance, Franettovich Smith, Coates [46] proposed that the elastic tape’s recoil properties could potentially offer mechanical support or opposition (such as deceleration or acceleration) to movement. This, in turn, could alleviate the strain on musculotendinous units by decreasing the load demands, while simultaneously allowing for the execution of a complete range of motion. Also, proprioceptive enhancement due to taping is identified as an effective means of prophylaxis to prevent injury [47, 48]. In this context, the improved proprioception and the resistive properties of athletic and Kinesiotape, may also help prevent movement into potentially injury-prone ranges ultimately reducing further risk of injury [32, 47, 49, 50]. Moreover, patellar taping has demonstrated non-biomechanical neurological effects that enhance motor performance [10, 51, 52]. An fMRI study revealed heightened activity in the cerebellum, primary somatosensory cortex, supplementary motor area, primary sensory cortex, and cingulate motor area due to taping [51]. The authors posited that the increased activation observed was indicative of improvements in coordination, sensation, decision-making, and coordination of non-conscious aspects of proprioception [51]. Similarly, recent electrophysiological data indicated that taping might facilitate proprioception by desynchronizing waves in the β-band in the motor cortex, aiding in precise force control during movement execution [52].

Conventionally, clinical assessment of proprioceptive acuity is conducted by tests evaluating kinematic and kinetic aspects of movement [53, 54]. The kinematic assessment by means of joint re-positioning accuracy measurement, the threshold to detection of passive motion, and active movement extent discrimination accuracy are widely evaluated in the existing literature [54]. However, the kinetic assessment of proprioception through force sense accuracy measurement has received less attention [55–57]. Force sense accuracy refers to a person’s active ability to sense and reproduce muscle tension or force [57, 58]. The assessment of force sense accuracy involves selecting a specific target force level, such as 50% of maximum voluntary isometric contraction and instructing the participant to reproduce this level of force during a muscle contraction at a specific joint angle [59, 60]. The discrepancy between the force produced by the participant and the target force level is then calculated and reported as the error in the sense of force. In the existing literature, the control of force sense has been hypothesized to exist due to an interplay between central feed-forward and peripheral feedback mechanisms [57]. Simon, Garcia [57] suggested that peripheral feedback mechanisms dominate during the initial phases of learning, whereas this dominance switches to feed-forward pathways upon gaining task-specific expertise [61]. In injuries when inherent proprioceptive accuracy is low, a larger dependence on peripheral feedback mechanisms is expected [62, 63]. Under such circumstances, the use of taping to facilitate force reproduction via peripheral feedback mechanisms makes sense. Docherty and Arnold [58] suggested that any improvement in force reproduction might signify an enhanced ability of a muscle to support the joint during movement, thereby reducing its predisposition towards injury [64].

Despite mounting evidence suggesting the beneficial influence of taping on proprioception and its gaining popularity, a lack of consensus exists in the literature regarding its efficacy, specifically concerning force sense accuracy. This lack of consensus exists primarily at the level of individual clinical trials. For instance, some individual trials have suggested the beneficial influence of taping on improving force sense accuracy [49, 65–67], whereas others have indicated that taping does not affect force sense accuracy [52, 57, 68]. Besides, several other outcomes need to be evaluated to determine the overall influence of taping on force sense accuracy outcomes. For instance, there is a need to evaluate the impact of taping on force sense from both between- and within-group perspectives. These findings could be significant because the analyses between the groups can provide insights into the contrasting results observed between taping and both the absence of a comparator and placebo taping. In contrast, the within-group analyses could explain the magnitude of change in proprioceptive parameters before and after the taping. Moreover, it is also important to classify the influence of taping in different population groups (i.e., healthy individuals and individuals with musculoskeletal/ neurological disorders). Evaluating this outcome is essential to understand the effectiveness of taping in various health conditions and could be helpful for both clinicians and patient population groups. Likewise, it is also crucial to assess separately the influence of different tapes on force sense accuracy. Taping interventions (i.e., Leuko, Kinesio, athletic, Zinc oxide tape) reported in the literature have been found to exhibit different elastic properties [69]. The literature suggests that different mechanisms are involved in modulating movement kinematics due to a change in the tape’s elasticity [46, 70]. Therefore, it makes sense that the influence of tapes is separately evaluated.

### Research aims and questions

1. To investigate the effects of taping on force sense accuracy in terms of absolute and relative accuracy from between- and within-group analyses.
2. To conduct exploratory subgroup meta-analyses of individual studies to investigate the potential impact of various factors such as study design, health status, taping elasticity on force sense accuracy.

## Material and methods

We followed PRISMA 2020 guidelines to conduct this systematic review and meta-analysis [71]. The checklist is presented in Supplementary Table S1. This systematic review was pre-registered at PROSPERO (CRD42022383616).

### Sources of data and search strategy

The systematic literature search was carried out across seven databases (Web of Science, PEDro, Pubmed, EBSCO, Scopus, EMBASE, Psychinfo) and one registry (Cochrane Central Register of Controlled Trials) from January 1946 until December 2022. These databases were chosen based on access provided by the academic organization. The search strategies according to each database have been provided in the Supplementary file. Furthermore, the authors conducted an extra search through the bibliography section of the relevant studies.

### Inclusion criteria

The inclusion criteria for the studies to be included in the review were developed according to the PICOS approach (Population, intervention, comparator, outcome of interest, and study design). The inclusion criteria were determined by two researchers (S.G, I.G). The inclusion criteria were as follows:

1. Incorporation of studies involving healthy individuals.
2. Inclusion of studies involving population groups affected by musculoskeletal disorders (such as a sprain, strain, tendinitis, repeated stress injuries, degenerative joint diseases, and traumatic injuries).
3. Inclusion of studies involving population groups affected by neurological disorders (such as stroke, Parkinson’s disease, cerebral palsy, multiple sclerosis, traumatic injuries, degenerative neurological disorders).
4. Inclusion of studies that evaluated the influence of taping on force sense accuracy. This encompassed various forms of taping, including tape, Kinesiotape, leuko tape, orthotic tape, adhesive tape, and others.
5. Inclusion of studies that assessed proprioception acuity through the analysis of active and relative force sense accuracy.
6. Inclusion of studies that compared taping intervention outcomes with a control group utilizing placebo tape and/or no tape.
7. Inclusion of all types of quantitative clinical study designs such as, randomized controlled trials, controlled clinical trials, crossover trials, longitudinal studies, cohort analyses, feasibility studies.
8. Inclusion of studies that achieved more than or equal to 4 on the PEDro quality appraisal scale [72].
9. Inclusion of studies published in peer-reviewed academic journals, theses, and conference proceedings.
10. Inclusion of studies published in English, French, German, or Hindi languages.

The intent behind maintaining a broad scope for the inclusion criteria was to ensure a comprehensive exploration of the subject matter. For example, the decision to encompass individuals with and without medical conditions stemmed from the goal of thoroughly investigating the potential variations in the effects of taping based on differing health statuses. Similarly, in acknowledging the diversity of study designs in existing research, we recognized the absence of a singular dominant design. By incorporating various study designs, our aim was to encompass a wider array of evidence and insights. Finally, the inclusion of diverse types of tapes was intended to scrutinize the potential distinct effects of these interventions based on their inherent elastic properties.

### Selection and extraction of data

The screening of the titles, abstracts, and full texts of the articles were conducted independently by two authors (S.G, I.G). During the screening process, both the authors were blinded from each other. In cases where there were disagreements about the inclusion of pertinent articles, the third author (S.N) participated in discussions to facilitate consensus. The following information was extracted from the articles: author names, country of research, participant information (age, sample size, gender distribution, health status), assessed joint, taping method, taping technique, application by physiotherapist, assessment periods, taping frequency, and results.

### Evaluation of the methodological quality

The PEDro quality appraisal scale was used to assess the quality of the studies included in our review [73]. The appraisal by the PEDro scale can be interpreted as follows: studies scoring between 9 to 11 are categorized as “excellent quality”, those with scores between 6 to 8 are considered “good quality”, scores between 4 to 5 indicate “fair quality”, and scores equal to or less than 3 signify “poor quality”. The appraisal of the studies was carried out by two researchers (S.G, I.G) independently. In instances of discrepancies, the researchers engaged in discussions, and if a unanimous decision couldn’t be reached, a third researcher (S.N) was consulted to achieve a consensus.

### Data analysis

In the present review, a between-group (taping vs. no comparator, taping vs. placebo taping) and a within-group (pre- vs. post-taping) random effect meta-analysis was conducted with Comprehensive meta-analysis (V 4.0) [74]. For the between-group analysis, we utilized mean change scores (i.e., post–pre performance outcomes) extracted from the respective studies. The data extracted from the studies were separately distributed and analyzed for force sense, encompassing active and relative accuracy. The meta-analysis outcomes reported comprise weighted and adjusted effect sizes (Hedge’s g), 95% confidence intervals (C.I.), and significance levels. The threshold for the interpretation of effect size were as follows: 0.16 denoted a small effect, 0.38 indicated a medium effect, and 0.76 signified a large effect [75]. Forest plots were generated to illustrate the results. Besides, the included studies’ heterogeneity was quantified using I^2^ statistics. The threshold for interpreting the heterogeneity with I^2^ statistics were defined as follows: between 0 and 40% indicated negligible heterogeneity, 30% to 60% denoted moderate heterogeneity, 50% to 90% represented substantial heterogeneity, and 75% to 100% indicated considerable heterogeneity [76]. In the present study, subgroup analyses were conducted based on study design (i.e., repeated measures design, quasi experimental studies), health status (i.e., healthy individuals, individuals with chronic ankle instability), and elasticity of taping (i.e., elastic, rigid tapes). An assessment of publication bias for the primary outcome was carried out according to the trim and fill procedure by Duval and Tweedie [77]. Additionally, we also conducted “leave-one-out” sensitivity analyses to test the robustness of our findings. The leave-one-out method systematically removes each study from the meta-analysis and re-analyzes the data to assess the influence of individual studies on the overall results [78]. This helps to identify studies that may be driving the results and assess the robustness of the findings. The significance level for the study was set at 0.05.

### Included studies

**Fig. 1:**
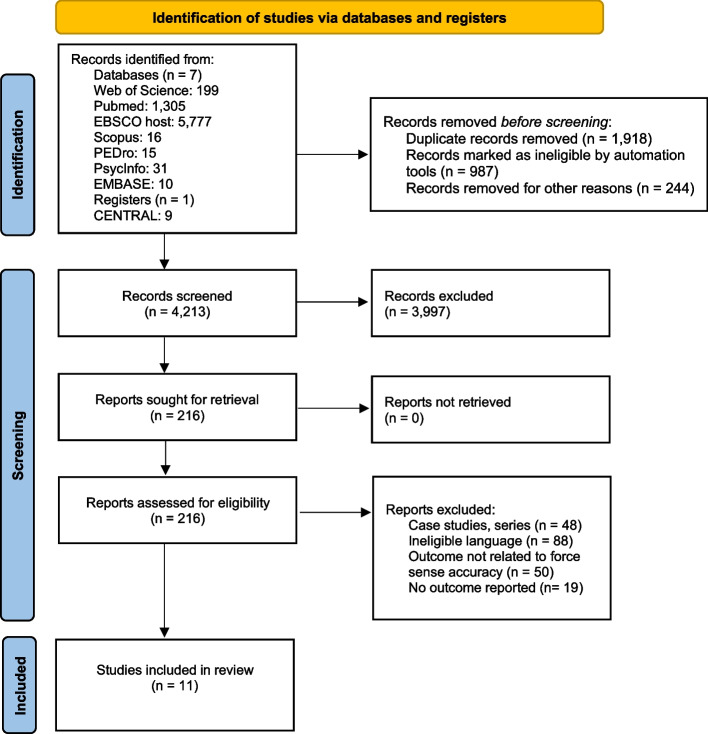
PRISMA flowchart of study selection (made from [79])

The initial search across seven databases and one registry yielded a total of 7362 articles, which after implementing the PICOS inclusion criteria, were reduced to 11 articles (Fig. 1). Thereafter, qualitative, and quantitative data were extracted from all included studies.

### Study design

Of the 11 included studies, five studies reported a repeated measures design [49, 52, 65, 80, 81], while four studies reported that they adhered to a case control format within a repeated measures design [57, 66, 82, 83]. Moreover, one study each reported to adhere to a pretest–posttest cross sectional design [68], and a pretest–posttest quasi-experimental design [67].

### Methodological quality

**Fig. 2:**
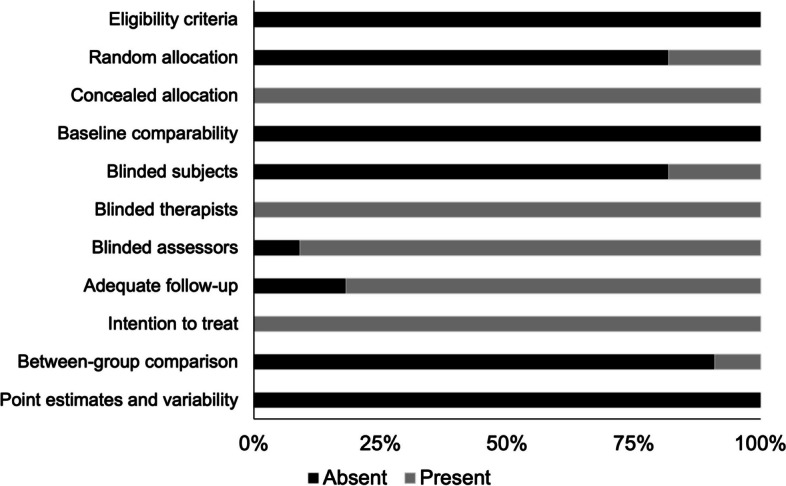
Illustrating the methodological quality according to the PEDro scale

The individual PEDro scoring for each included study has been tabulated in Table 1. The average PEDro quality score of the 11 included studies was (5.8 ± 0.9), suggesting the overall quality of the included studies to be “fair”. The two researchers (S.G, I.G) appraised the articles with a 97.3% agreement. In terms of individual scores, one study scored eight [68], seven studies received a score of 6 [49, 52, 57, 65, 66, 80, 82], two studies achieved a score of 5 [81, 83], and one studies attained a score of 4 [67]. The methodological quality across the studies has also been illustrated in Fig. 2.

**Table 1:** PEDro methodological quality for the included studies

	Total PEDro score	Point estimates and variability	Between-group comparison	Intention to treat	Adequate follow-up	Blinded assessors	Blinded therapists	Blinded subjects	Baseline comparability	Concealed allocation	Random allocation	Eligibility criteria
Li, Wei [81]	5	**-**	**-**	**+**	**+**	**+**	**+**	**+**	**-**	**+**	**-**	**-**
Lin, Yang [52]	6	**-**	**-**	**+**	**+**	**+**	**+**	**-**	**-**	**+**	**-**	**-**
Han [49]	6	**-**	**-**	**+**	**+**	**+**	**+**	**-**	**-**	**+**	**-**	**-**
Hosseini, Salehi Dehno [68]	8	**-**	**-**	**+**	**-**	**-**	**+**	**-**	**-**	**+**	**-**	**-**
Momeni-lari, Ghasemi [67]	4	**-**	**+**	**+**	**+**	**+**	**+**	**-**	**-**	**+**	**+**	**-**
Hopper, Grisbrook [80]	6	**-**	**-**	**+**	**+**	**+**	**+**	**-**	**-**	**+**	**-**	**-**
Simon, Garcia [57]	6	**-**	**-**	**+**	**-**	**+**	**+**	**-**	**-**	**+**	**+**	**-**
Chang, Cheng [66]	6	**-**	**-**	**+**	**+**	**+**	**+**	**-**	**-**	**+**	**-**	**-**
Chang, Wang [82]	6	**-**	**-**	**+**	**+**	**+**	**+**	**-**	**-**	**+**	**-**	**-**
Lee, Kwon [83]	5	**-**	**-**	**+**	**+**	**+**	**+**	**+**	**-**	**+**	**-**	**-**
Chang, Chou [65]	6	**-**	**-**	**+**	**+**	**+**	**+**	**-**	**-**	**+**	**-**	**-**

**+**: bias present, **-**: bias absent

### Publication bias

**Fig. 3:**
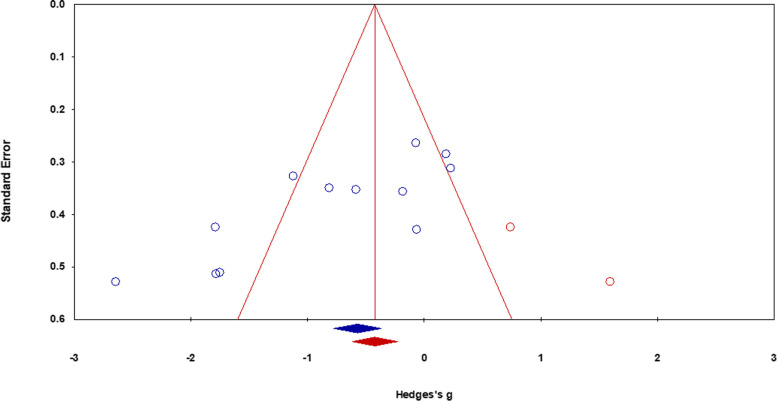
A trim and fill funnel plot illustrating the publication bias. Blue circle: individual studies, red circle: imputed studies, funnel plot: 95% area of the pseudo-confidence intervals, vertical midline: estimated overall effect size (i.e., empirical studies + imputed studies)

The incidence of publication bias according to Duval and Tweedie’s trim and fill procedure is shown in Fig. 3. The method identified two missing studies on the right side of the mean effect, whereas no study was missing on the left side. In the analysis, under the random effect model, the point estimate and the 95% C.I. for the combined studies was -0.79, -1.27 to -0.31. Based on the trim and fill procedure, the imputed point estimates are -0.54 (-1.04 to -0.05).

## Results

### Systematic review report

#### Participants

Among the 11 included studies, data from a total of 279 (128F, 94M) people was reported. The difference in sex distribution was because two studies had not reported these values for their sample [82, 83]. The average age of the entire sample was 24.5 ± 11.7 years. A comprehensive depiction of the health status of the participants featured in this review is provided in Table 2.

**Table 2:** Details of cohorts with different health statuses included in this review

**Health status classification**	**Additional subgroup information**	**Studies; references**	**Sample size (Female, Male)**	**Age**	**Studies not reporting descriptive; references**
Healthy	-	9; [49, 52, 57, 65, 66, 68, 80, 82, 83]	150 (90F, 60M)	27.9 ± 13.7	2; [82, 83]
Epicondylitis	Medial epicondylitis	2; [66, 82]	20 (10F)	19.5 ± 1.5	1; [82]
	Lateral epicondylitis	1; [83]	15 (?)	41.9 ± 6.8	1; [83]
Functional ankle instability	-	2; [57, 67]	34 (25F, 9M)	48.5 ± 4.8	-
	Fatigue	1; [84]	28 (13F, 15M)	21.2 ± 2	-

#### Countries

Four of the 11 studies were conducted in Taiwan [52, 65, 66, 82], two in South Korea [49, 83], two in Iran [67, 68], one in Australia [80], one in China [84], and one in USA [57].

#### Tape

There are three different types of tapes evaluated in the included studies. A total of nine studies used Kinesiotape [49, 52, 57, 65–68, 82, 84], one used Zinc oxide tape [83], and one used rigid sports tape [80]. As per established literature, Kinesiotape is categorized as an elastic tape [85], whereas zinc oxide and rigid sports tape are classified as rigid tapes [86].

#### Force sense accuracy assessment

The included studies reported absolute and relative force sense accuracy outcomes. All 11 included studies reported the absolute force sense accuracy values [49, 52, 57, 65–68, 80, 82–84], with an additional subset of five studies also offering insights into relative force sense accuracy values [65, 66, 68, 82, 84].

#### Joints assessed

Six of the included studies evaluated the efficacy of taping on the wrist joint [52, 65, 66, 68, 82, 83]. In contrast, only five studies reported the influence of taping on the ankle joint [49, 57, 67, 80, 84].

#### Taping application

Five of the included studies did not report the person who applied the tape [52, 65–68], and five reported that a physiotherapist applied the taping [49, 80, 82–84]. Furthermore, one study reported that a healthcare practitioner applied the tape [57].

#### Outcome

The details of the included studies and their respective outcomes are mentioned in Table 3.

**Table 3:** Details of the included studies

Study *Design*	Health status/ medical condition *Joint assessed*	Sample size (N) Gender distribution (F, M) (Age in years as Mean ± SD/ range)	Taping methods *Taping application by*	Force sense assessment	Taping technique	Timing of post-test	Results
Li, Wei [81] *Repeated measure design*	Functional ankle instability with fatigue *Ankle*	*N* = 28 13F, 15 M (21.2 ± 2)	F-KT: Facilitatory Kinesiotape AB-KT: Ankle balance Kinesiotape PT: Placebo tape NT: No tape *Physical therapist*	Absolute force sense reproduction at 25% of MVIC Variable force sense reproduction at 25% of MVIC	FKT: Applied according to Kase’s technique [25] in four strips with 50% tension, initial position with the ankle in slight plantarflexion Strip 1: Applied on anterior mid-foot (115–120% stretch), attached below anterior tibial tuberosity over tibialis anterior Strip 2: Applied above the medial malleolus, the wrapped around heel like a stirrup, attached just lateral to strip 1 Strip 3: Applied across the ankle anteriorly, covering both lateral and medial malleoli Strip 4: Applied on the arch with a slight stretch (4–6 inches) above both lateral and medial malleoli AB-KT: Applied according to [84] in four steps with 50% tension Step 1: Applied to perform posterior talar glide, the ankle was held in slight dorsiflexion while applying kinesiology tape from the talus to the calcaneus Step 2: Applied to perform ankle inversion, the ankle was held in slight inversion while applying kinesiology tape from 5 cm above the medial malleolus through the lateral calcaneus to the outside of the instep Step 3: Applied to perform ankle eversion, the ankle was held in slight eversion while applying kinesiology tape from 5 cm above the lateral malleolus through the medial calcaneus to the inside of the instep Step 4: Applied over the first tape, covering it completely to increase ankle support and posterior talar glide while holding the ankle in slight dorsiflexion. PT: Applied in two steps Step 1: Applied from below the medial calcaneus to midway up the medial aspect of the lower leg Step 2: Applied from below the lateral malleolus to midway up the lateral aspect of the lower leg	Post-fatigue F-KT/AB-KT/PT	No difference in absolute force sense accuracy with F-KT, AB-KT as compared to PT or NT No difference in relative variable force sense accuracy with F-KT, AB-KT as compared to PT or NT
Lin, Yang [52] *Repeated measures design*	Healthy *Wrist*	*N* = 24 12F, 12 M (22.9 ± 1.5)	KT: Kinesiotape KT 20%: Kinesiotape with 20% additional stretch NT: No tape *Not reported*	Absolute force sense reproduction at 20% of MVIC	KT: Y-shape strip applied on the skin (0% stretch) above finger flexors with an anchor set at the wrist, then KT extended to tendon region of finger flexor KT 20%: Similar to KT but tension applied on the middle area of tape (20% stretch), and no stress on the ends of the tape	Pre-KT/KT 20% Post- KT/KT 20%: -	No difference in absolute force sense accuracy with KT, KT 20% as compared to NT
Han [49] *Repeated measures design*	Healthy (geriatric) *Ankle*	*N* = 13 5F, 8 M 64.4 ± 6.95	KT: Kinesiotape PT: placebo tape NT: No tape *Physical Therapist*	Absolute force sense reproduction at 50% of MVIC	KT: Applied in an initial position with calf stretched according to [87] from the proximal gastrocnemius muscle insertion to calcaneus bone with tension (15–20% stretch), KT applied in four steps Step 1: KT anchored at heel with ankle joint in the neutral position Step 2: Calf muscle stretched Step 3: Y-strip divided the proximal end of the tape and then attached to the medial and lateral end of the gastrocnemius muscle Step 4: I-strip applied from the posterior surface of the calcaneus to the upper part of the gastrocnemius junction PT: KT was applied in three strips on the heel and medial, and lateral head of the gastrocnemius muscle	Pre-KT/PT Post-KT/PT: immediately after with KT/PT	Significant ↑ in absolute force sense accuracy with KT as compared to PT and NT
Hosseini, Salehi Dehno [68] *Cross-sectional pretest–posttest design*	Healthy *Wrist*	KT OI: *N* = 15 15F (24.9 ± 3.7) KT IO: *N* = 15 15F (24.9 ± 3.7) NT: *N* = 15 15F (24.9 ± 3.7)	KT OI: Kinesiotape from origin to insertion KT OI: Kinesiotape from insertion to origin NT: No tape *Not reported*	Absolute force sense reproduction at 50% of MVIC Relative force sense reproduction at 50% of MVIC	KT: Applied on the wrist flexor muscles of the dominant hand with 30% tension according to Kase’s procedure [25] KT sliced from the middle to produce two tails of a Y-strip and applied on the anterior part of the forearm with the arm relaxed and in supination and the wrist, and elbow in full extension KT OI: Base of Y-strip applied near medial epicondyle of humerus and two tails positioned around muscle belly, the two distal parts were attached around the base of the fifth metacarpal KT IO: Base of Y-strip applied near fifth metacarpal and two tails positioned around muscle belly, the two proximal parts were attached around medial epicondyle of humerus	Pre-KT Post-KT: 24 h after	Significant ↑ in relative force sense accuracy in KT OI group as compared to NT No difference in absolute force sense accuracy 24 h after KT OI, KT IO as compared to NT No difference in relative force sense accuracy 24 h after KT IO as compared to NT
Momeni-lari, Ghasemi [67] *Pretest–posttest Quasi-experimental design*	Functional ankle instability *Ankle*	*N* = 20 20F (27.7 ± 8.1)	KT: Kinesiotape *Not reported*	Absolute force sense reproduction at 50% of MVIC	KT: Applied with two I-strips and one Y-strip with tendon correction technique I-strip 1: Applied (50% stretch) from anterior tibialis anterior in plantar flexion and eversion from the middle of the leg to the tibial tuberosity I-strip 2: Applied (50% stretch) for gastrocnemius in dorsiflexion from posterior ankle to knee joint Y-strip 1: Applied (50% stretch) for peroneus in dorsiflexion and inversion from the outer ankle surface to the back of the head of the fibula	Pre-KT Post-KT: immediately after KT	Significant ↑ in absolute force sense accuracy with KT
Hopper, Grisbrook [80] *Repeated measures design*	Healthy *Ankle*	*N* = 16 16F (22.9 ± 3.9)	RT: Rigid sports tape NT: No tape *Physical Therapist*	Absolute force sense reproduction at 30% of MVIC	RT: Applied on the ankle joint to support medial and lateral ligament complex while allowing complete range of motion with hindfoot taping technique from Hopper, McNair [88] RT applied in three steps Step 1: two stirrups applied Step 2: stirrups were followed by two half eight Step 3: RT finished with a horizontal locking tape	Pre-RT Post-RT: immediately after RT	No difference in absolute force sense accuracy with RT as compared to NT
Simon, Garcia [57] *Case control repeated measures design*	Functional ankle instability *Ankle*	*N* = 14 5F, 9 M (20.8 ± 1.4)	KT: Kinesiotape *Healthcare provider*	Absolute force sense reproduction at 30% of MVIC	KT: Four strips were applied according to Kase’s procedure [89] Strip 1: applied on the dorsum of the foot and then up the anterior aspect of the ankle and the lower leg ending distal to the knee approximately over the tibial tuberosity Strip 2: applied on the plantar surface of the foot and traveled laterally over the lateral malleolus and lateral aspect of the lower leg, the strip ended on the proximal lower leg over the head of the fibula Strip 3: applied anteriorly across the ankle from the medial to the lateral aspect Strip 4: applied on the plantar surface of the foot anterior to the second strip, it then proceeded laterally and ended on the anteromedial aspect of the lower leg approximately one-third of the way up the leg	Pre-KT Post-KT: immediately after, 72 h after	No difference in absolute force sense accuracy immediately after, 72 h after KT
	Healthy *Ankle*	*N* = 14 12F, 2 M (21.2 ± 2.6)	NT: No tape *-*		-	-	No difference in absolute force sense accuracy immediately after, 72 h after KT
Chang, Cheng [66] *Case control repeated measures design*	Medial epicondylitis *Wrist*	*N* = 10 10 M (19.5 ± 1.5)	KT: Kinesiotape PT: Placebo tape NT: No tape *Not reported*	Absolute force sense reproduction at 50% of MVIC Relative force sense reproduction at 50% of MVIC	KT: Applied on the wrist flexor muscles of the dominant hand according to Kase’s procedure [25] To ensure the stretch of tape equals 15% to 20%, stretch KT was cut from the middle to produce a Y-strip. Y-strip applied on common wrist flexor muscle from its insertion to origin. The first tail of the Y-strip was applied on the middle part of the forearm with the wrist in hyperextension, the elbow in full extension, and the forearm in full supination. The second tail of the Y-strip was applied from insertion to origin along the medial edge of the forearm to wrap common wrist flexor muscles	Pre-KT Post-KT/PT: immediately after KT/PT	Significant ↑ in absolute force sense accuracy with KT as compared to NT No difference in absolute force sense accuracy with KT as compared to PT No difference in relative force sense accuracy with KT as compared to PT and NT
	Healthy *Wrist*	*N* = 17 17 M (19.9 ± 1.5)					Significant ↑ in absolute force sense accuracy with KT as compared to NT No difference in absolute force sense accuracy with KT as compared to PT No difference in relative force sense accuracy with KT as compared to PT and NT
Chang, Wang [82] *Case control repeated measures design*	Medial epicondylitis *Wrist*	*N* = 10 ? (19.5 ± 1.4)	KT: Kinesiotape PT: Placebo tape NT: No tape *Physical Therapist*	Absolute force sense reproduction at 50% of MVIC Relative force sense reproduction at 50% of MVIC	KT: Applied on the wrist flexor muscles of the dominant hand according to Kase’s procedure [25] To ensure the stretch of tape equals 15% to 20%, stretch Kt was cut from the middle to produce a Y-strip. Y-strip applied on common wrist flexor muscle from its insertion to origin. The first tail of the Y-strip was applied on the middle part of the forearm with the wrist in hyperextension, the elbow in full extension, and the forearm in full supination. The second tail of the Y-strip was applied from insertion to origin along the medial edge of the forearm to wrap common wrist flexor muscles	Pre-KT Post-KT/PT: immediately after KT/PT	No difference in absolute force sense accuracy between KT, NT and PT No difference in relative force sense accuracy with KT as compared to PT and NT
	Healthy *Wrist*	*N* = 17 ? (19.8 ± 1.5)					Significant ↑ in absolute force sense accuracy with KT as compared to NT and PT No difference in relative force sense accuracy with KT as compared to PT and NT Significant ↑ in relative force sense accuracy with PT as compared to NT
Lee, Kwon [83] *Case control repeated measures design*	Lateral epicondylitis *Wrist*	*N* = 15 ?F, ?M (41.9 ± 6.8)	ZnOT: Zinc oxide tape NT: No tape *Physical Therapist*	Absolute active RE during wrist extension for target angle 20º, 25º, 30º	ZnOT: Applied with the wrist extended to contract with extensor carpi radialis brevis, then tape applied on the proximal forearm starting medially and tracking laterally, the process repeated twice or thrice The tape tightened as per the subject’s tolerability and was snug during the contraction of wrist extensors	Pre-ZnOT Post-ZnOT: Immediately after ZnOT	No difference in absolute force sense accuracy with ZnOT as compared to NT
	Healthy *Wrist*	*N* = 15 ?F, ?M (42 ± 5.2)					No difference in absolute force sense accuracy with ZnOT as compared to NT
Chang, Chou [65] *Repeated measures design*	Healthy *Wrist*	*N* = 21 21 M (20.8 ± 2.6)	KT: Kinesiotape PT: Placebo tape NT: No tape *Not reported*	Absolute force sense reproduction at 50% of MVIC Relative force sense reproduction at 50% of MVIC	KT: Applied on the wrist flexor muscles of the dominant hand according to Kase’s procedure [25] Y-strip applied on common wrist flexor muscle from its insertion to origin with 15% to 20% stretch. The first tail of the Y-strip was applied on the middle part of the forearm with the wrist in hyperextension, the elbow in full extension, and the forearm in full supination. The second tail of the Y-strip was applied from insertion to origin with a 15% to 20% stretch along the medial edge of the forearm PT: KT applied as an I-strip with 0% stretch, applied from the inferior region to the medial epicondyle of the humerus from the middle line of the medial side of the forearm and across the belly of the common wrist flexor with 15% to 20% stretch to wrap common wrist flexor muscles	Pre-KT Post-KT/PT: immediately after KT/PT	Significant ↑ in absolute force sense accuracy with KT as compared to PT and NT Significant ↑ in relative force sense accuracy with KT as compared to PT and NT

*MVIC* Maximum voluntary isometric contraction, *F* Female, *M* Male

#### Outcomes based on type of comparator

##### Taping vs. no comparator

Absolute accuracy: Nine studies compared the efficacy of taping intervention with no taping [49, 52, 65, 66, 68, 80–83]. Two studies reported significant improvement in force sense accuracy outcomes with taping compared to the absence of a comparator [49, 65]. Additionally, one study reported a significant improvement in force sense accuracy outcomes within their sample of healthy individuals, but not among individuals with medial epicondylitis [82]. Conversely, another study reported a significant improvement in force sense accuracy outcomes within their sample of individuals with medial epicondylitis, but not among healthy individuals [66]. Moreover, five studies did not report any significant differences between the taping intervention and the absence of a comparator [52, 68, 80, 81, 83].

Relative accuracy: Three studies had compared the efficacy of taping intervention with no taping [66, 68, 82]. Two studies reported no significant difference between the taping and the no taping group [66, 82]. One study reported a significant improvement in force sense accuracy between their origin to insertion taping subgroup as compared to the no taping group [68]. However, no significant differences were observed between insertion to origin taping and no taping comparator [68].

##### Taping vs. placebo taping

Absolute accuracy: Five studies had compared the efficacy of taping intervention with placebo taping [49, 65, 66, 81, 82]. Among these, two studies reported a significant improvement in force sense accuracy outcomes with taping compared to the placebo comparator [49, 65]. One study indicated a significant improvement in force sense accuracy outcomes within their sample of healthy individuals but not among those with medial epicondylitis [82]. Furthermore, two studies did not report any significant difference between taping and the placebo comparator [66, 81].

Relative accuracy: Two studies compared the efficacy of taping intervention with a placebo comparator [66, 82]. Neither of these studies found a significant difference between the taping intervention and the placebo comparator.

##### Pre vs. post differences

Three studies evaluated the within group differences after the application of the taping intervention [57, 67, 83]. Among these, two studies reported no significant differences in force sense accuracy after the application of taping [57, 83], while one study reported a significant improvement in force sense accuracy after the taping application [67].

### Meta-analysis report

**Fig. 4:**
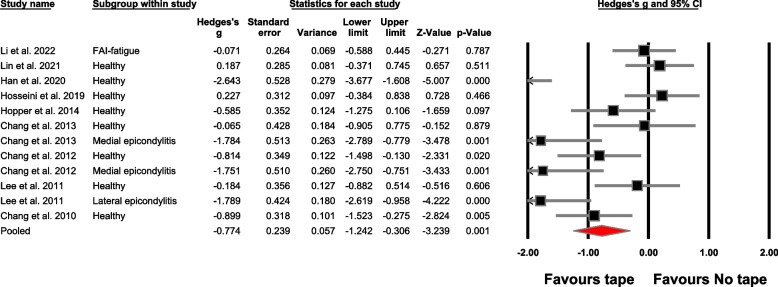
Forest plot in this study illustrates the effect of taping on absolute force sense improvement in accuracy. Black boxes: individual weighted effect sizes, whiskers: 95% confidence intervals, red diamond: pooled weighted effect size and 95% confidence interval, positive effect size: improvement in force sense accuracy for the no-taping group, negative effect size: improvement in force sense accuracy for the taping group

**Fig. 5:**
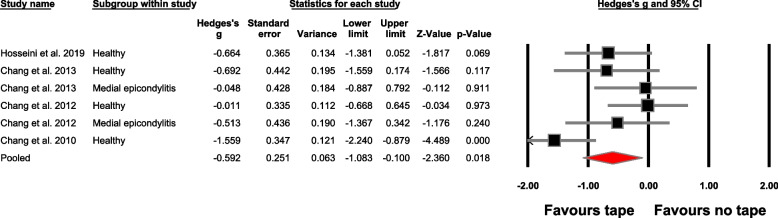
Forest plot illustrating the effect of taping on relative force sense accuracy. Black boxes: individual weighted effect sizes, whiskers: 95% confidence intervals, red diamond: pooled weighted effect size and 95% confidence interval, positive effect size: improvement in force sense accuracy for the no-taping group, negative effect size: improvement in force sense accuracy for the taping group

**Fig. 6:**
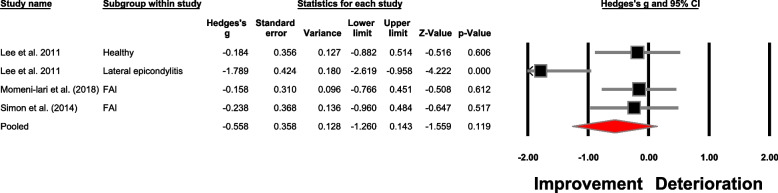
Forest plot illustrating the within-group effect of taping on force sense improvement in accuracy. Black boxes: individual weighted effect sizes, whiskers: 95% confidence intervals, red diamond: pooled weighted effect size and 95% confidence interval, positive effect size: deterioration in force sense accuracy, negative effect size: improvement in force sense accuracy. (FAI: Functional ankle instability)

The meta-analysis findings are detailed in Table 4, Figs. 4 and 5, offering an extensive perspective on the between-group analysis. Similarly, Table 5 and Fig. 6 visually depict the within-group meta-analysis outcomes. Additionally, Table 6 provides an in-depth report on the leave one out sensitivity analysis.

**Table 4:** Between-group meta-analysis outcomes

**No.**	**Outcome**	**Number of studies; (References)**	**Meta-analysis outcome** Hedge’s g, 95% Confidence interval, *p*-value	**Heterogeneity** I^2^	**Figure**
**Absolute force accuracy (comparator: no tape)**					
1	Overall	9; [49, 52, 65, 66, 68, 80–83]	-0.77 (-1.24 to -0.30), *p* = 0.001	80%	Fig. 4
Study design					
2	Repeated measures design^a^	8; [49, 52, 65, 66, 80–83]	-0.87 (-1.36 to -0.38), *p* < 0.001	80%	S1
3	Cross-sectional design	1; [68]	-	-	-
Tape type					
4	Elastic tape	7; [49, 52, 65, 66, 68, 81, 82]	-0.76 (-1.33 to -0.19), *p* = 0.009	83%	S2
5	Rigid tape	2; [80, 83]	-0.82 (-1.71 to 0.06), *p* = 0.069	77%	S3
Population group					
6	Healthy	8; [49, 52, 65, 66, 68, 82, 83]	-0.53 (-1.05 to -0.01), *p* = 0.044	78%	S4
7	Medial epicondylitis	2; [66, 82]	-1.76 (-2.47 to -1.05), *p* < 0.001	0%	S5
8	Lateral epicondylitis	1; [83]	-	-	-
9	Functional ankle instability with fatigue	1; [81]	-	-	-
Population group and tape type					
10	Healthy (elastic tapes)	6; [49, 52, 65, 66, 68, 82]	-0.60 (-1.31 to 0.10), *p* = 0.092	84%	S6
11	Healthy (rigid tapes)	2; [80, 83]	-0.38 (-0.87 to 0.10), *p* = 0.12	0%	S7
12	Medial epicondylitis (elastic tape)	Same as outcome number 7			
13	Lateral epicondylitis (rigid tape)	1; [83]	-	-	-
14	Functional ankle instability with fatigue (elastic tape)	1; [81]	-	-	-
**Relative force accuracy (comparator: no tape)**					
15	Overall	4; [65, 66, 68, 82]	-0.59 (-1.08 to -0.10), *p* = 0.018	60%	Fig. 5
Study design					
16	Repeated measures design^a^	3; [65, 66, 82]	-0.57 (-1.18 to 0.03), *p* = 0.065	68%	S8
17	Cross-sectional design	1; [68]	-	-	-
Tape type					
18	Elastic tape	Same as outcome number 15			
19	Rigid tape	-	-	-	-
Population group					
20	Healthy	4; [65, 66, 68, 82]	-0.73 (-1.40 to -0.058), *p* = 0.03	71%	S9
21	Medial epicondylitis	2; [66, 82]	-0.27 (-0.87 to 0.32), *p* = 0.36	0%	S10
Population group (tape type)					
22	Healthy (elastic tape)	Same as outcome number 20			
23	Medial epicondylitis (elastic tape)	Same as outcome number 21			
**Absolute accuracy (comparator: Placebo tape)**					
24	Overall	5; [49, 65, 66, 81, 82]	-0.51 (-0.91 to -0.10), *p* = 0.01	55%	S11
Study design					
25	Repeated measures design^a^	Same as outcome number 24			
Tape type					
26	Elastic tape	Same as outcome number 24			
27	Rigid tape	-	-	-	-
Population group					
28	Healthy	4; [49, 65, 66, 82]	-0.76 (-1.32 to -0.21), *p* = 0.007	56%	S12
29	Medial epicondylitis	2; [66, 82]	-0.30 (-0.90 to 0.29), *p* = 0.31	0%	S13
30	Functional ankle instability-fatigue	1; [81]	-	-	-
Population group (tape type)					
31	Healthy (elastic tape)	Same as outcome number 28			
32	Medial epicondylitis (elastic tape)	Same as outcome number 29			
33	Functional ankle instability-fatigue (elastic tape)	1; [81]	-	-	-
**Relative accuracy (comparator: Placebo tape)**					
34	Overall	3; [65, 66, 82]	0.50 (-1.15 to 0.15), *p* = 0.13	72%	S14
Study design					
35	Repeated measures design^a^	Same as outcome number 34			
Tape type					
36	Elastic tape	Same as outcome number 34			
37	Rigid tape	-	-	-	-
Population group					
38	Healthy	3; [65, 66, 82]	-0.76 (-1.73 to 0.20), *p* = 0.12	81%	S15
39	Medial epicondylitis	2; [66, 82]	-0.06 (-0.66 to 0.52), *p* = 0.82	0%	S16
Population group (tape type)					
40	Healthy (elastic tape)	Same as outcome number 38			
41	Medial epicondylitis (elastic tape)	Same as outcome number 39			

^a^Including studies with case control repeated measures design

**Table 5:** Within-group meta-analysis outcomes

**No.**	**Outcome**	**Number of studies; (References)**	**Meta-analysis outcome** Hedge’s g, 95% Confidence interval, *p*-value	**Heterogeneity** I^2^	**Figure**
**Absolute force sense accuracy**					
42	Overall	3; [57, 67, 83]	-0.55 (-1.26 to 0.14), *p* = 0.11	74%	Fig. 6
Study design					
43	Repeated measures design^a^	2; [57, 83]	-0.71 (-1.68 to 0.26), *p* = 0.15	80%	S17
44	Quasi experimental design	1; [67]	-	-	-
Tape type					
45	Elastic tape	2; [57, 67]	-0.19 (-0.65 to 0.27), *p* = 0.42	0%	S18
46	Rigid tape	1; [83]	-	-	-
Population group					
47	Functional ankle instability	Same as outcome number 45			
48	Healthy	1; [83]	-	-	-
49	Lateral epicondylitis	1; [83]	-	-	-
Population group (tape type)					
50	Functional ankle instability (elastic tape)	Same as outcome number 45			
51	Healthy (rigid tape)	1; [83]	-	-	-
52	Lateral epicondylitis (rigid tape)	1; [83]	-	-	-

^a^Including studies with case control repeated measures design

**Table 6:** Leave one out sensitivity analysis

**No.**	**Analysis**	**Meta-analysis** ***p*-value**	**I**^**2**^	**Studies impacting the *p*-value upon removal**	***P*-value upon removal**	**Figure**
**Between group (Absolute force accuracy: no comparator)**						
1.	Overall	0.001	80%	-	No effect	S19
2.	Repeated measures design^a^	< 0.001	80%	-	No effect	S20
3.	Cross-sectional design	-	-	-	-	-
4.	Elastic tape	0.009	83%	-	No effect	S21
5.	Rigid tape	0.069	77%	-	No effect	S22
6.	Healthy	0.044	78%	Han [49]	0.109	S23
				Hopper, Grisbrook [80]	0.080	
				Chang, Wang [82]	0.096	
				Lee, Kwon [83]	0.051	
				Chang, Chou [65]	0.104	
7.	Medial epicondylitis	< 0.001	0%	-	No effect	S24
8.	Lateral epicondylitis	-	-	-	-	-
9.	Functional ankle instability with fatigue	-	-	-	-	-
10.	Healthy (elastic tapes)	0.092	84%	-	No effect	S25
11.	Healthy (rigid tape)	0.123	**0%**	-	-	S26
12.	Medial epicondylitis (elastic tape)	Same as outcome number 7				
13.	Lateral epicondylitis (rigid tape)	-	-	-	-	-
14.	Functional ankle instability with fatigue (elastic tape)	-	-	-	-	-
**Between group (Relative force accuracy: no comparator)**						
15.	Overall	0.018	60%	Hosseini, Salehi Dehno [68]	0.065	S27
				Chang, Cheng [66]	0.056	
16.	Repeated measures design^a^	0.065	68%	Chang, Wang [82] (healthy)	0.032	S28
17.	Cross-sectional design	-	-	-	-	-
18.	Elastic tape	Same as outcome number 15				
19.	Rigid tape	-	-	-	-	-
20.	Healthy	0.03	71%	Hosseini, Salehi Dehno [68]	0.069	S29
				Chang, Cheng [66]	0.117	
				Chang, Wang [82]	0.973	
21.	Medial epicondylitis	0.36	0%	-	No effect	S30
22.	Healthy (elastic tape)	Same as outcome number 20				
23.	Medial epicondylitis (elastic tape)	Same as outcome number 21				
**Between group (Absolute accuracy: placebo comparator)**						
24.	Overall	0.01	55%	Chang, Chou [65]	0.056	S31
25.	Repeated measures design^a^	Same as outcome number 24				
26.	Elastic tape	Same as outcome number 24				
27.	Rigid tape	-	**-**	-	-	-
28.	Healthy	0.007	56%	Chang, Chou [65]	0.089	S32
29.	Medial epicondylitis	0.31	0%	-	No effect	S33
30.	Functional ankle instability (fatigue)	-	-	-	-	-
31.	Healthy (elastic tape)	Same as outcome number 28				
32.	Medial epicondylitis (elastic tape)	Same as outcome number 29				
33.	Functional ankle instability-fatigue (elastic tape)	-	-	-	-	-
**Between group (Relative accuracy: placebo comparator)**						
34.	Overall	0.13	72%	-	No effect	S34
35.	Repeated measures design^a^	Same as outcome number 34				
36.	Elastic tape	Same as outcome number 34			-	-
37.	Rigid tape	-	-	-	-	-
38.	Healthy	0.12	81%	-	No effect	S35
39.	Medial epicondylitis	0.82	0%	-	No effect	S36
40.	Healthy (elastic tape)	Same as outcome number 38				
41.	Medial epicondylitis (elastic tape)	Same as outcome number 39				
**Within group (Absolute force accuracy)**						
42.	Overall	0.11	74%	-	No effect	S37
43.	Repeated measures design^a^	0.15	80%	-	No effect	S38
44.	Quasi experimental design	-	-	-	-	-
45.	Elastic tape	0.42	0%	-	No effect	S39
46.	Rigid tape	-	-	-	-	-
47.	Functional ankle instability	Same as outcome number 45				
48.	Healthy	-	-	-	-	-
49.	Lateral epicondylitis	-	-	-	-	-
50.	Functional ankle instability (elastic tape)	Same as outcome number 45				
51.	Healthy (rigid tape)	-	-	-	-	-
52.	Lateral epicondylitis (rigid tape)	-	-	-	-	-

^a^Including studies with case control repeated measures design

### Sensitivity analysis

A summary of the leave-one-out sensitivity analysis has been provided in Table 6. Specifically, studies were reported if the overall analysis yielded a *p*-value less than 0.05, and the removal of a specific study increased the *p*-value above this threshold. Conversely, studies were also reported if the overall analysis yielded a *p*-value greater than 0.05 and the removal of any study decreased the *p*-value below this threshold.

## Discussion

The current systematic review and meta-analysis aimed to comprehensively evaluate the effect of taping on force sense accuracy in healthy and patient population groups. Our meta-analysis confirms these findings, as we observed a significant effect improvement in both absolute (Hedge’s g: -0.77) and relative (g: -0.59) force sense accuracy with taping as compared to no comparator in the between-group analysis. Moreover, we also observed a significant improvement in absolute (g: -0.51) force sense accuracy with taping as compared to placebo taping. However, the within-group analysis revealed a non-significant small effect improvement in absolute force sense accuracy. The sub-sections below have discussed our further subgroup analyses concerning various population groups and tape types.

### Influence of taping on different population groups

Our findings suggest that augmentation of proprioceptive afferent by taping is more beneficial for individuals with poorer inherent proprioception than individuals with good proprioception. The reason being that taping augmented proprioceptive afferent information might overload the “inherently good” proprioceptive pathways in healthy individuals [33]. In contrast, individuals with poorer proprioception (i.e., medial epicondylitis) might benefit from augmented afferent information [90, 91]. We believe two main reasons might explain this differential result in our study. First, there was a large difference in the number of studies in our meta-analysis that included different population groups. For instance, in the between-group analysis, the influence of taping was evaluated on healthy individuals within eight studies. In comparison, only two studies evaluated taping’s impact on individuals with medial epicondylitis. Second, in the analyses of healthy population groups, we did not conduct separate sub-group analyses to assess the differential influence on individuals with excellent and poor inherent proprioception (i.e., inherent force sense accuracy). The analysis was not performed because only one included study had classified their healthy cohort based on their innate proprioceptive levels [52]. Future studies are strongly recommended to organize the proprioceptive level of their population groups to help understand the actual influence of taping on force sense accuracy among healthy individuals.

Furthermore, taping improved force sense accuracy in population groups with injuries, such as individuals with medial epicondylitis, lateral epicondylitis, and functional ankle instability. One potential explanation for the improved force sense accuracy in this sample is that taping may have restricted the injured musculoskeletal tissue to its anatomical limits, leading to an enhanced sense of force at the joint [32]. The work of Seo also supports this hypothesis, who suggested that taping allows injured ligaments to heal in their proper position, reducing the risk of chronic instability, while non-taped injuries may heal in a stretched, weakened state [92]. Simon, Garcia [57] demonstrated that Kinesiotaping compensated for deficits in force sense accuracy in individuals with functional ankle instability (pre-tape: 2.6N, post-tape: 2.2N, 72 h: 1.8N). The authors hypothesized that the Kinesiotape could have allowed their sample to establish a new perceptual trace that allowed them to process the afferent information more efficiently, thereby improving their ability to sense force [57]. Chang, Wang [82] too reported the beneficial influence of taping among individuals with medial epicondylitis. The authors hypothesized that Kinesiotape could have alleviated deficits in force sense reproduction by stimulating the mechanoreceptors in the skin and fascia to perceive better changes in shear forces, stretch, pressure, and load [82, 93]. In this context, the central nervous system could have utilized this improved perception facilitated by taping to fine-tune the feed-forward models. This, in turn, contributed to an augmentation in the accuracy of force sensation [57, 82]. Besides, one of our included studies also suggested that perhaps the increments in force sense resulted from the pain-relieving properties of taping [83]. Studies have indicated that because of noxious muscle input, the excitability at the motor cortex level is reduced together with the inhibition of spinal motor neurons [94, 95]. Under such circumstances, taping could have modulated the pain through the pain gait control mechanism [96]. This could involve an increased flow of sensory signals from low-threshold peripheral mechanoreceptors, which might have countered the transmission of pain signals to central nociceptive cells, ultimately leading to a reduction in pain perception [97, 98]. Additionally, the observation of significant improvement in force sense accuracy with actual taping as compared to no taping, as well as taping as compared to placebo taping, suggests that the effects of taping might go beyond the realm of a simple placebo effect. While the placebo effect of taping can indeed influence an individual’s perception and performance [99], the fact that actual taping shows a more pronounced improvement indicates that there might be additional physiological mechanisms at play. Nevertheless, the conclusive determination of taping’s true effects, potentially extending beyond placebo responses, necessitates future studies.

### Influence of taping elasticity on force sense accuracy

Various tapes have been used in the existing literature to influence force sense accuracy outcomes in healthy and patient population groups. Some studies have directly compared the influence of different types of tapes on proprioceptive results [45, 100, 101]. However, uncertainty still looms regarding which tape is the most effective. The literature suggests that tapes with lower elastic modulus (i.e., high rigidity) function primarily by limiting the range of motion at a joint rather than facilitating its neuromuscular functioning [102, 103]. Rigid tapes are suggested to be beneficial for individuals who need additional stability to participate in certain activities or to protect an injured area.

Nonetheless, the restrictive nature of such rigid tapes has been suggested to predispose someone to higher risks of injury, as the tape can alter joint kinematics, such as excessive talocrural movement restriction [104]. Besides, as these tapes lack sufficient adhesive capabilities to cope with functional activities, their use is repeatedly questioned in sporting and rehabilitation contexts [101, 105]. On the contrary, tapes with high elastic modulus have been reported to support and stabilize the joints without restricting the joint’s range of motion. Due to their increased flexibility, elastic tapes can conform to the body’s contours, allowing for a greater range of motion. This elasticity can be beneficial specifically for individuals who need to perform functional activities. A study included in our review suggested that Kinesiotape differed from conventional rigid tapes in addition to the enhanced elasticity as it also embedded a specialized wave-like grain design [65]. According to the authors, this specialized design and the tape’s elasticity could have facilitated proprioception by exerting a pulling force on the skin while generating space by lifting the underlying fascia and soft tissue [25, 65].

In our between-group meta-analyses, we observed that elastic tapes (*p* = 0.009) led to a significantly improved absolute force sense accuracy, whereas rigid tapes did not show a significant effect (*p* = 0.069). Furthermore, when we performed subgroup analyses considering health status, we found a greater degree of enhancement, although not statistically significant, in the accuracy of absolute force sensing among healthy individuals who used elastic tape (effect size: -0.60) compared to those who used rigid tape (effect size: -0.38). We did not perform a comparative analysis on the effectiveness of rigid tape in reducing relative force sense accuracy due to limited available data. Nevertheless, several potential explanations for the observed variance in the degree of force sense accuracy between elastic and rigid tapes can be considered. Firstly, it is possible that healthy individuals who used elastic tape did not require the restrictive support provided by rigid tapes but benefited from the functional support offered by elastic tapes, which improved joint movement and proprioception. Secondly, the smaller number of studies included in the analysis of rigid tapes (only two) compared to elastic tape (six) may have contributed to greater variability in the data or an increased risk of a type II error. Therefore, we recommend that future studies be conducted with high-quality trials and sufficient sample sizes to further investigate and compare the efficacy of elastic and rigid tapes in more detail.

### Limitations

The main goal of our study was to understand the impact of taping on force sense accuracy. However, our analysis had some limitations. Some studies included in our review evaluated the immediate effects of taping [49, 65–67, 82, 83], while others examined the impact of prolonged taping on force sense accuracy [57, 68]. As our study did not precisely aim to explore the effect of prolonged taping on proprioception, we did not perform separate analyses to compare the impact of prolonged taping on force sense accuracy. Studies have shown that prolonged taping may have a greater effect on proprioception’s kinematic and kinetic aspects than taping applied immediately [106–108]. Therefore, future studies are strongly recommended to evaluate the differential influence of the prolonged application of taping on force sense accuracy. Secondly, substantial heterogeneity was also prominent regarding the different taping application methods. This heterogeneity could be an essential aspect the reader should consider while interpreting the results. For instance, some studies included in our review adhered to a specific technique, such as Kenzo Kase’s technique [57, 65, 66, 68, 82], whereas the majority had applied taping without following any standardized approach [49, 52, 67, 80]. This heterogeneous approach to using tape complicates the understanding regarding which method of taping is more influential in improving force sense accuracy. Therefore, we recommend that future studies adhere to standardized taping applications as they can help develop practical, evidence-based guidelines. Thirdly, despite employing a broad inclusion criterion, we did not come across any high-quality, blinded, randomized controlled trials that had assessed the impact of taping on force sense accuracy. The absence of randomized controlled trials in our analysis could potentially raise concerns about the reliability and validity of our findings. Consequently, we strongly recommend that future studies further expand upon our current findings by evaluating the effects of taping interventions on force sense accuracy through well-designed, high-quality, blinded randomized controlled trials. Fourthly, while our primary objective was to assess how taping affects accuracy parameters in force sensing, we did not analyze its impact on other kinetic measures of maximum voluntary contraction, such as dynamometry and surface electromyography. Exploring the effects of taping on these measures could have provided additional insights relating to the heterogeneity of the results. We recommend that future reviews to examining the impact of taping on other kinetic indicators as the findings from these studies will enhance our comprehension of how taping influences proprioceptive control related to force. Another major limitation of our study was that fewer studies were included in our meta-analyses, such as between-group analyses of individuals with medial epicondylitis (i.e., two studies) and within-group analyses of individuals with functional ankle instability (two studies). Fewer studies could increase the chances of a type II error [109]. Again, the reader is advised to infer these results with caution.

### Future directions

Although the number of studies incorporating taping for improving proprioception in healthy and patient population groups has increased in the past decade, a few aspects still warrant exploration. For instance, limited research has evaluated the long-term retention of force sense reproduction after the application of taping [57, 68]. Conventionally, taping has been identified as a transient approach that facilitates performance transiently by guiding the movement when it is being worn. However, once its removed, the lack of guidance (see guidance hypothesis [110]) by taping forces improved accuracy back to initial levels [111]. We presume that an effective means by which this feedback dependency of taping could be countered by tapering the extent of tactile feedback provided over time. Here, perhaps reducing the length of taping applied [112], or even the tension with which taping is used, could reduce the extent of feedback being provided to the performer and allow them to form robust internal feed-forward models concerning the task at hand. Future studies should try to evaluate these outcomes to ascertain whether tapering the feedback by taping can promote learning in terms of force sense reproduction compared to performance.

## Conclusions

Our exploratory meta-analysis of a limited number of studies reports the positive influence of taping on absolute force sense accuracy outcomes when compared to no compared to both no taping and placebo taping. The improvements in relative force sense accuracy was only significant with taping when compared to no taping. Collectively, the advancement in accuracy for both relative and absolute force sensing through taping potentially indicates the potential for improved performance and a lowered risk of injury. Specifically, the improved accuracy in perceiving relative force might facilitate precise fine-tuning of force while engaging in various activities, while the enhanced absolute force perception could suggest accurate application of force. This heightened precision becomes particularly valuable in dynamic high-risk environments where maintaining balance and executing complex movements are necessary.

Moreover, we observed a significant improvement in force sense accuracy for elastic tapes as compared to rigid tapes for absolute force sense accuracy. Unfortunately, due to the predominance of studies with a repeated measures design within our pooled data, and the relatively fewer studies employing cross-sectional or quasi-experimental designs, a comprehensive comparative analysis based on study design could not be performed. Lastly, healthy individuals and individuals with medial epicondylitis were observed to significantly improve their absolute force sense accuracy with taping when compared to taping, as compared to no taping. However, when compared to placebo taping a significant improvement in force sense accuracy was only observed for healthy individuals.

Despite the sensitivity analyses confirming the robustness of our findings, we recommend our reader interpret these results cautiously as the studies included in our review were of “fair” methodological quality, and high levels of heterogeneity were observed in our meta-analyses. Future studies are recommended to further evaluate the efficacy of taping on force sense accuracy outcomes in high-quality randomized controlled trials to ascertain the true effects of taping on force sense accuracy outcomes.

### Supplementary Information

**Additional file 1: Table S1.** PRISMA checklist. **Figure S1.** Forest plot illustrating the effect of taping on absolute force sense accuracy (repeated measures design). Black boxes: individual weighted effect sizes, whiskers: 95% confidence intervals, red diamond: pooled weighted effect size and 95% confidence interval, positive effect size: improved force sense accuracy for the no-taping group, negative effect size: improved force sense accuracy for the taping group. **Figure S2.** Forest plot illustrating the effect of taping on absolute force sense accuracy (elastic tape). Black boxes: individual weighted effect sizes, whiskers: 95% confidence intervals, red diamond: pooled weighted effect size and 95% confidence interval, positive effect size: improved force sense accuracy for the no-taping group, negative effect size: improved force sense accuracy for the elastic taping group. **Figure S3.** Forest plot illustrating the effect of taping on absolute force sense accuracy (rigid tape). Black boxes: individual weighted effect sizes, whiskers: 95% confidence intervals, red diamond: pooled weighted effect size and 95% confidence interval, positive effect size: improved force sense accuracy for the no-taping group, negative effect size: improved force sense accuracy for the rigid taping group. **Figure S4.** Forest plot illustrating the effect of taping on absolute force sense accuracy (healthy individuals). Black boxes: individual weighted effect sizes, whiskers: 95% confidence intervals, red diamond: pooled weighted effect size and 95% confidence interval, positive effect size: improved force sense accuracy for the no-taping group, negative effect size: improved force sense accuracy for the taping group. **Figure S5.** Forest plot illustrating the effect of taping on absolute force sense accuracy (individuals with medial epicondylitis). Black boxes: individual weighted effect sizes, whiskers: 95% confidence intervals, red diamond: pooled weighted effect size and 95% confidence interval, positive effect size: improved force sense accuracy for the no-taping group, negative effect size: improved force sense accuracy for the taping group. **Figure S6.** Forest plot illustrating the effect of taping on absolute force sense accuracy (healthy individuals with elastic tape). Black boxes: individual weighted effect sizes, whiskers: 95% confidence intervals, red diamond: pooled weighted effect size and 95% confidence interval, positive effect size: improved force sense accuracy for the no-taping group, negative effect size: improved force sense accuracy for the elastic taping group. **Figure S7.** Forest plot illustrating the effect of taping on absolute force sense accuracy (healthy individuals with rigid tape). Black boxes: individual weighted effect sizes, whiskers: 95% confidence intervals, red diamond: pooled weighted effect size and 95% confidence interval, positive effect size: improved force sense accuracy for the no-taping group, negative effect size: improved force sense accuracy for the rigid taping group. **Figure S8.** Forest plot illustrating the effect of taping on relative force sense accuracy (repeated measures design). Black boxes: individual weighted effect sizes, whiskers: 95% confidence intervals, red diamond: pooled weighted effect size and 95% confidence interval, positive effect size: improved force sense accuracy for the no-taping group, negative effect size: improved force sense accuracy for the taping group. **Figure S9.** Forest plot illustrating the effect of taping on relative force sense accuracy (healthy individuals). Black boxes: individual weighted effect sizes, whiskers: 95% confidence intervals, red diamond: pooled weighted effect size and 95% confidence interval, positive effect size: improved force sense accuracy for the no-taping group, negative effect size: improved force sense accuracy for the taping group. **Figure S10.** Forest plot illustrating the effect of taping on relative force sense accuracy (individuals with medial epicondylitis). Black boxes: individual weighted effect sizes, whiskers: 95% confidence intervals, red diamond: pooled weighted effect size and 95% confidence interval, positive effect size: improved force sense accuracy for the no-taping group, negative effect size: improved force sense accuracy for the taping group. **Figure S11.** Forest plot illustrating the effect of taping on absolute force sense accuracy. Black boxes: individual weighted effect sizes, whiskers: 95% confidence intervals, red diamond: pooled weighted effect size and 95% confidence interval, positive effect size: improved force sense accuracy for the placebo taping group, negative effect size: improved force sense accuracy for the taping group. **Figure S12.** Forest plot illustrating the effect of taping on absolute force sense accuracy (healthy individuals). Black boxes: individual weighted effect sizes, whiskers: 95% confidence intervals, red diamond: pooled weighted effect size and 95% confidence interval, positive effect size: improved force sense accuracy for the placebo taping group, negative effect size: improved force sense accuracy for the taping group. **Figure S13.** Forest plot illustrating the effect of taping on absolute force sense accuracy (individuals with medial epicondylitis). Black boxes: individual weighted effect sizes, whiskers: 95% confidence intervals, red diamond: pooled weighted effect size and 95% confidence interval, positive effect size: improved force sense accuracy for the placebo taping group, negative effect size: improved force sense accuracy for the taping group. **Figure S14.** Forest plot illustrating the effect of taping on relative force sense accuracy. Black boxes: individual weighted effect sizes, whiskers: 95% confidence intervals, red diamond: pooled weighted effect size and 95% confidence interval, positive effect size: improved force sense accuracy for the placebo taping group, negative effect size: improved force sense accuracy for the taping group. **Figure S15.** Forest plot illustrating the effect of taping on relative force sense accuracy (healthy individuals). Black boxes: individual weighted effect sizes, whiskers: 95% confidence intervals, red diamond: pooled weighted effect size and 95% confidence interval, positive effect size: improved force sense accuracy for the placebo taping group, negative effect size: improved force sense accuracy for the taping group. **Figure S16.** Forest plot illustrating the effect of taping on relative force sense accuracy (individuals with medial epicondylitis). Black boxes: individual weighted effect sizes, whiskers: 95% confidence intervals, red diamond: pooled weighted effect size and 95% confidence interval, positive effect size: improved force sense accuracy for the placebo taping group, negative effect size: improved force sense accuracy for the taping group. **Figure S17.** Forest plot illustrating the effect of taping on absolute force sense accuracy (repeated measures design). Black boxes: individual weighted effect sizes, whiskers: 95% confidence intervals, red diamond: pooled weighted effect size and 95% confidence interval, positive effect size: deterioration in force sense accuracy, negative effect size: improvement in force sense accuracy. **Figure S18.** Forest plot illustrating the effect of taping on absolute force sense accuracy (elastic tape). Black boxes: individual weighted effect sizes, whiskers: 95% confidence intervals, red diamond: pooled weighted effect size and 95% confidence interval, positive effect size: deterioration in force sense accuracy, negative effect size: improvement in force sense accuracy. **Figure S19.** Leave-one-out sensitivity analysis for between group analysis for absolute force sense accuracy (taping vs. no comparator). Black boxes: individual weighted effect sizes, whiskers: 95% confidence intervals, red diamond: pooled weighted effect size and 95% confidence interval, positive effect size: improvement in force sense accuracy for the no-taping group, negative effect size: improvement in force sense accuracy for the taping group. **Figure S20.** Leave-one-out sensitivity analysis for between group analysis for absolute force sense accuracy (repeated measures design, taping vs. no comparator). Black boxes: individual weighted effect sizes, whiskers: 95% confidence intervals, red diamond: pooled weighted effect size and 95% confidence interval, positive effect size: improvement in force sense accuracy for the no-taping group, negative effect size: improvement in force sense accuracy for the taping group. **Figure S21.** Leave-one-out sensitivity analysis for between group analysis for absolute force sense accuracy (elastic tape, elastic taping vs. no comparator). Black boxes: individual weighted effect sizes, whiskers: 95% confidence intervals, red diamond: pooled weighted effect size and 95% confidence interval, positive effect size: improvement in force sense accuracy for the no-taping group, negative effect size: improvement in force sense accuracy for the elastic taping group. **Figure S22.** Leave-one-out sensitivity analysis for between group analysis for absolute force sense accuracy (rigid tape, rigid taping vs. no comparator). Black boxes: individual weighted effect sizes, whiskers: 95% confidence intervals, red diamond: pooled weighted effect size and 95% confidence interval, positive effect size: improvement in force sense accuracy for the no-taping group, negative effect size: improvement in force sense accuracy for the rigid taping group. **Figure S23.** Leave-one-out sensitivity analysis for between group analysis for absolute force sense accuracy (healthy individuals, taping vs. no comparator). Black boxes: individual weighted effect sizes, whiskers: 95% confidence intervals, red diamond: pooled weighted effect size and 95% confidence interval, positive effect size: improvement in force sense accuracy for the no-taping group, negative effect size: improvement in force sense accuracy for the taping group. **Figure S24.** Leave-one-out sensitivity analysis for between group analysis for absolute force sense accuracy (individuals with medial epicondylitis, taping vs. no comparator). Black boxes: individual weighted effect sizes, whiskers: 95% confidence intervals, red diamond: pooled weighted effect size and 95% confidence interval, positive effect size: improvement in force sense accuracy for the no-taping group, negative effect size: improvement in force sense accuracy for the taping group. **Figure S25.** Leave-one-out sensitivity analysis for between group analysis for absolute force sense accuracy (healthy individuals, elastic taping vs. no comparator). Black boxes: individual weighted effect sizes, whiskers: 95% confidence intervals, red diamond: pooled weighted effect size and 95% confidence interval, positive effect size: improvement in force sense accuracy for the no-taping group, negative effect size: improvement in force sense accuracy for the elastic taping group. **Figure S26.** Leave-one-out sensitivity analysis for between group analysis for absolute force sense accuracy (healthy individuals, rigid taping vs. no comparator). Black boxes: individual weighted effect sizes, whiskers: 95% confidence intervals, red diamond: pooled weighted effect size and 95% confidence interval, positive effect size: improvement in force sense accuracy for the no-taping group, negative effect size: improvement in force sense accuracy for the rigid taping group. **Figure S27.** Leave-one-out sensitivity analysis for between group analysis for relative force sense accuracy (taping vs. no comparator). Black boxes: individual weighted effect sizes, whiskers: 95% confidence intervals, red diamond: pooled weighted effect size and 95% confidence interval, positive effect size: improvement in force sense accuracy for the no-taping group, negative effect size: improvement in force sense accuracy for the taping group. **Figure S28.** Leave-one-out sensitivity analysis for between group analysis for relative force sense accuracy (repeated measures design, taping vs. no comparator). Black boxes: individual weighted effect sizes, whiskers: 95% confidence intervals, red diamond: pooled weighted effect size and 95% confidence interval, positive effect size: improvement in force sense accuracy for the no-taping group, negative effect size: improvement in force sense accuracy for the taping group. **Figure S29.** Leave-one-out sensitivity analysis for between group analysis for relative force sense accuracy (healthy individuals, taping vs. no comparator). Black boxes: individual weighted effect sizes, whiskers: 95% confidence intervals, red diamond: pooled weighted effect size and 95% confidence interval, positive effect size: improvement in force sense accuracy for the no-taping group, negative effect size: improvement in force sense accuracy for the taping group. **Figure S30.** Leave-one-out sensitivity analysis for between group analysis for relative force sense accuracy (individuals with medial epicondylitis, taping vs. no comparator). Black boxes: individual weighted effect sizes, whiskers: 95% confidence intervals, red diamond: pooled weighted effect size and 95% confidence interval, positive effect size: improvement in force sense accuracy for the no-taping group, negative effect size: improvement in force sense accuracy for the taping group. **Figure S31.** Leave-one-out sensitivity analysis for between group analysis for absolute force sense accuracy (taping vs. placebo taping comparator). Black boxes: individual weighted effect sizes, whiskers: 95% confidence intervals, red diamond: pooled weighted effect size and 95% confidence interval, positive effect size: improvement in force sense accuracy for the placebo taping group, negative effect size: improvement in force sense accuracy for the taping group. **Figure S32.** Leave-one-out sensitivity analysis for between group analysis for absolute force sense accuracy (healthy individuals, taping vs. placebo taping comparator). Black boxes: individual weighted effect sizes, whiskers: 95% confidence intervals, red diamond: pooled weighted effect size and 95% confidence interval, positive effect size: improvement in force sense accuracy for the placebo taping group, negative effect size: improvement in force sense accuracy for the taping group. **Figure S33.** Leave-one-out sensitivity analysis for between group analysis for absolute force sense accuracy (individuals with medial epicondylitis, taping vs. placebo taping comparator). Black boxes: individual weighted effect sizes, whiskers: 95% confidence intervals, red diamond: pooled weighted effect size and 95% confidence interval, positive effect size: improvement in force sense accuracy for the placebo taping group, negative effect size: improvement in force sense accuracy for the taping group. **Figure S34.** Leave-one-out sensitivity analysis for between group analysis for relative force sense accuracy (taping vs. placebo taping comparator). Black boxes: individual weighted effect sizes, whiskers: 95% confidence intervals, red diamond: pooled weighted effect size and 95% confidence interval, positive effect size: improvement in force sense accuracy for the placebo taping group, negative effect size: improvement in force sense accuracy for the taping group. **Figure S35.** Leave-one-out sensitivity analysis for between group analysis for relative force sense accuracy (healthy individuals, taping vs. placebo taping comparator). Black boxes: individual weighted effect sizes, whiskers: 95% confidence intervals, red diamond: pooled weighted effect size and 95% confidence interval, positive effect size: improvement in force sense accuracy for the placebo taping group, negative effect size: improvement in force sense accuracy for the taping group. **Figure S36.** Leave-one-out sensitivity analysis for between group analysis for relative force sense accuracy (individuals with medial epicondylitis, taping vs. placebo taping comparator). Black boxes: individual weighted effect sizes, whiskers: 95% confidence intervals, red diamond: pooled weighted effect size and 95% confidence interval, positive effect size: improvement in force sense accuracy for the placebo taping group, negative effect size: improvement in force sense accuracy for the taping group. **Figure S37.** Leave-one-out sensitivity analysis for within group analysis for absolute force sense accuracy. Black boxes: individual weighted effect sizes, whiskers: 95% confidence intervals, red diamond: pooled weighted effect size and 95% confidence interval, positive effect size: deterioration in force sense accuracy, negative effect size: improvement in force sense accuracy. **Figure S38.** Leave-one-out sensitivity analysis for within group analysis for absolute force sense accuracy (repeated measures design). Black boxes: individual weighted effect sizes, whiskers: 95% confidence intervals, red diamond: pooled weighted effect size and 95% confidence interval, positive effect size: deterioration in force sense accuracy, negative effect size: improvement in force sense accuracy. **Figure S39.** Leave-one-out sensitivity analysis for within group analysis for absolute force sense accuracy (elastic tape). Black boxes: individual weighted effect sizes, whiskers: 95% confidence intervals, red diamond: pooled weighted effect size and 95% confidence interval, positive effect size: deterioration in force sense accuracy, negative effect size: improvement in force sense accuracy. **Search strategy for individual databases**.

## Data Availability

The datasets used and/or analysed during the current study are available from the corresponding author on reasonable request.
