# Supplementary material for: Influence of taping on force sense accuracy: a systematic review with between and within group meta-analysis
Source: BMC Sports Sci Med Rehabil. 2023 Oct 20;15:138. doi: 10.1186/s13102-023-00740-1 (PMC10588111; doi:10.1186/s13102-023-00740-1)
Supplement: Supplementary file 1 — Additional file 1: Table S1. PRISMA checklist. Figure S1. Forest plot illustrating the effect of taping on absolute force sense accuracy (repeated measures design). Black boxes: individual weighted effect sizes, whiskers: 95% confidence intervals, red diamond: pooled weighted effect size and 95% confidence interval, positive effect size: improved force sense accuracy for the no-taping group, negative effect size: improved force sense accuracy for the taping group. Figure S2. Forest plot illustrating the effect of taping on absolute force sense accuracy (elastic tape). Black boxes: individual weighted effect sizes, whiskers: 95% confidence intervals, red diamond: pooled weighted effect size and 95% confidence interval, positive effect size: improved force sense accuracy for the no-taping group, negative effect size: improved force sense accuracy for the elastic taping group. Figure S3. Forest plot illustrating the effect of taping on absolute force sense accuracy (rigid tape). Black boxes: individual weighted effect sizes, whiskers: 95% confidence intervals, red diamond: pooled weighted effect size and 95% confidence interval, positive effect size: improved force sense accuracy for the no-taping group, negative effect size: improved force sense accuracy for the rigid taping group. Figure S4. Forest plot illustrating the effect of taping on absolute force sense accuracy (healthy individuals). Black boxes: individual weighted effect sizes, whiskers: 95% confidence intervals, red diamond: pooled weighted effect size and 95% confidence interval, positive effect size: improved force sense accuracy for the no-taping group, negative effect size: improved force sense accuracy for the taping group. Figure S5. Forest plot illustrating the effect of taping on absolute force sense accuracy (individuals with medial epicondylitis). Black boxes: individual weighted effect sizes, whiskers: 95% confidence intervals, red diamond: pooled weighted effect size and 95% confidence interval [file 13102_2023_740_MOESM1_ESM.docx]

**Influence of taping on force sense accuracy: A systematic review with between and within group meta-analysis**

Supplementary file

**Table S1** PRISMA checklist

| **Section and Topic** | **Item #** | **Checklist item** | **Location where item is reported** |
| --- | --- | --- | --- |
| **TITLE** | | |  |
| Title | 1 | Identify the report as a systematic review. | 1 |
| **ABSTRACT** | | |  |
| Abstract | 2 | See the PRISMA 2020 for Abstracts checklist. | 1 |
| **INTRODUCTION** | | |  |
| Rationale | 3 | Describe the rationale for the review in the context of existing knowledge. | 1-3 |
| Objectives | 4 | Provide an explicit statement of the objective(s) or question(s) the review addresses. | 3 |
| **METHODS** | | |  |
| Eligibility criteria | 5 | Specify the inclusion and exclusion criteria for the review and how studies were grouped for the syntheses. | 3-4 |
| Information sources | 6 | Specify all databases, registers, websites, organisations, reference lists and other sources searched or consulted to identify studies. Specify the date when each source was last searched or consulted. | 3 |
| Search strategy | 7 | Present the full search strategies for all databases, registers and websites, including any filters and limits used. | 3, Supplementary file Page 19 |
| Selection process | 8 | Specify the methods used to decide whether a study met the inclusion criteria of the review, including how many reviewers screened each record and each report retrieved, whether they worked independently, and if applicable, details of automation tools used in the process. | 4 |
| Data collection process | 9 | Specify the methods used to collect data from reports, including how many reviewers collected data from each report, whether they worked independently, any processes for obtaining or confirming data from study investigators, and if applicable, details of automation tools used in the process. | 4 |
| Data items | 10a | List and define all outcomes for which data were sought. Specify whether all results that were compatible with each outcome domain in each study were sought (e.g. for all measures, time points, analyses), and if not, the methods used to decide which results to collect. | 4 |
|  | 10b | List and define all other variables for which data were sought (e.g. participant and intervention characteristics, funding sources). Describe any assumptions made about any missing or unclear information. | 4 |
| Study risk of bias assessment | 11 | Specify the methods used to assess risk of bias in the included studies, including details of the tool(s) used, how many reviewers assessed each study and whether they worked independently, and if applicable, details of automation tools used in the process. | 4 |
| Effect measures | 12 | Specify for each outcome the effect measure(s) (e.g. risk ratio, mean difference) used in the synthesis or presentation of results. | 4 |
| Synthesis methods | 13a | Describe the processes used to decide which studies were eligible for each synthesis (e.g. tabulating the study intervention characteristics and comparing against the planned groups for each synthesis (item #5)). | 4 |
|  | 13b | Describe any methods required to prepare the data for presentation or synthesis, such as handling of missing summary statistics, or data conversions. | 4 |
|  | 13c | Describe any methods used to tabulate or visually display results of individual studies and syntheses. | 4 |
|  | 13d | Describe any methods used to synthesize results and provide a rationale for the choice(s). If meta-analysis was performed, describe the model(s), method(s) to identify the presence and extent of statistical heterogeneity, and software package(s) used. | 4 |
|  | 13e | Describe any methods used to explore possible causes of heterogeneity among study results (e.g. subgroup analysis, meta-regression). | 4 |
|  | 13f | Describe any sensitivity analyses conducted to assess robustness of the synthesized results. | 4 |
| Reporting bias assessment | 14 | Describe any methods used to assess risk of bias due to missing results in a synthesis (arising from reporting biases). | 4 |
| Certainty assessment | 15 | Describe any methods used to assess certainty (or confidence) in the body of evidence for an outcome. | NA |
| **RESULTS** | | |  |
| Study selection | 16a | Describe the results of the search and selection process, from the number of records identified in the search to the number of studies included in the review, ideally using a flow diagram. | 4-5 |
|  | 16b | Cite studies that might appear to meet the inclusion criteria, but which were excluded, and explain why they were excluded. | - |
| Study characteristics | 17 | Cite each included study and present its characteristics. | 4-13 |
| Risk of bias in studies | 18 | Present assessments of risk of bias for each included study. | 5-7 |
| Results of individual studies | 19 | For all outcomes, present, for each study: (a) summary statistics for each group (where appropriate) and (b) an effect estimates and its precision (e.g. confidence/credible interval), ideally using structured tables or plots. | 4-13 |
| Results of syntheses | 20a | For each synthesis, briefly summarise the characteristics and risk of bias among contributing studies. | 5-7 |
|  | 20b | Present results of all statistical syntheses conducted. If meta-analysis was done, present for each the summary estimate and its precision (e.g. confidence/credible interval) and measures of statistical heterogeneity. If comparing groups, describe the direction of the effect. | 14-17 |
|  | 20c | Present results of all investigations of possible causes of heterogeneity among study results. | 14-17 |
|  | 20d | Present results of all sensitivity analyses conducted to assess the robustness of the synthesized results. | 14, 18-19 |
| Reporting biases | 21 | Present assessments of risk of bias due to missing results (arising from reporting biases) for each synthesis assessed. | 5, 7 |
| Certainty of evidence | 22 | Present assessments of certainty (or confidence) in the body of evidence for each outcome assessed. | - |
| **DISCUSSION** | | |  |
| Discussion | 23a | Provide a general interpretation of the results in the context of other evidence. | 14, 19-20 |
|  | 23b | Discuss any limitations of the evidence included in the review. | 19-20 |
|  | 23c | Discuss any limitations of the review processes used. | 19-20 |
|  | 23d | Discuss implications of the results for practice, policy, and future research. | 20-21 |
| **OTHER INFORMATION** | | |  |
| Registration and protocol | 24a | Provide registration information for the review, including register name and registration number, or state that the review was not registered. | 3 |
|  | 24b | Indicate where the review protocol can be accessed, or state that a protocol was not prepared. | Protocol was not prepared |
|  | 24c | Describe and explain any amendments to information provided at registration or in the protocol. | In the registration the main outcome was referred as force sense error, while in the manuscript it was referred to as force sense accuracy. Moreover, some search keywords had to be modified according to individual databases during the search. |
| Support | 25 | Describe sources of financial or non-financial support for the review, and the role of the funders or sponsors in the review. | 22, Funders played no role in the review. |
| Competing interests | 26 | Declare any competing interests of review authors. | - |
| Availability of data, code and other materials | 27 | Report which of the following are publicly available and where they can be found: template data collection forms; data extracted from included studies; data used for all analyses; analytic code; any other materials used in the review. | - |

*From:*  Page MJ, McKenzie JE, Bossuyt PM, Boutron I, Hoffmann TC, Mulrow CD, et al. The PRISMA 2020 statement: an updated guideline for reporting systematic reviews. BMJ 2021;372:n71. doi: 10.1136/bmj.n71

For more information, visit: <http://www.prisma-statement.org/>

**Figure S1**. Forest plot illustrating the effect of taping on absolute force sense accuracy (repeated measures design). Black boxes: individual weighted effect sizes, whiskers: 95% confidence intervals, red diamond: pooled weighted effect size and 95% confidence interval, positive effect size: improved force sense accuracy for the no-taping group, negative effect size: improved force sense accuracy for the taping group.

**Figure S2**. Forest plot illustrating the effect of taping on absolute force sense accuracy (elastic tape). Black boxes: individual weighted effect sizes, whiskers: 95% confidence intervals, red diamond: pooled weighted effect size and 95% confidence interval, positive effect size: improved force sense accuracy for the no-taping group, negative effect size: improved force sense accuracy for the elastic taping group.

**Figure S3**. Forest plot illustrating the effect of taping on absolute force sense accuracy (rigid tape). Black boxes: individual weighted effect sizes, whiskers: 95% confidence intervals, red diamond: pooled weighted effect size and 95% confidence interval, positive effect size: improved force sense accuracy for the no-taping group, negative effect size: improved force sense accuracy for the rigid taping group.

**Figure S4**. Forest plot illustrating the effect of taping on absolute force sense accuracy (healthy individuals). Black boxes: individual weighted effect sizes, whiskers: 95% confidence intervals, red diamond: pooled weighted effect size and 95% confidence interval, positive effect size: improved force sense accuracy for the no-taping group, negative effect size: improved force sense accuracy for the taping group.

**Figure S5**. Forest plot illustrating the effect of taping on absolute force sense accuracy (individuals with medial epicondylitis). Black boxes: individual weighted effect sizes, whiskers: 95% confidence intervals, red diamond: pooled weighted effect size and 95% confidence interval, positive effect size: improved force sense accuracy for the no-taping group, negative effect size: improved force sense accuracy for the taping group.

**Figure S6**. Forest plot illustrating the effect of taping on absolute force sense accuracy (healthy individuals with elastic tape). Black boxes: individual weighted effect sizes, whiskers: 95% confidence intervals, red diamond: pooled weighted effect size and 95% confidence interval, positive effect size: improved force sense accuracy for the no-taping group, negative effect size: improved force sense accuracy for the elastic taping group.

**Figure S7**. Forest plot illustrating the effect of taping on absolute force sense accuracy (healthy individuals with rigid tape). Black boxes: individual weighted effect sizes, whiskers: 95% confidence intervals, red diamond: pooled weighted effect size and 95% confidence interval, positive effect size: improved force sense accuracy for the no-taping group, negative effect size: improved force sense accuracy for the rigid taping group.

**Figure S8**. Forest plot illustrating the effect of taping on relative force sense accuracy (repeated measures design). Black boxes: individual weighted effect sizes, whiskers: 95% confidence intervals, red diamond: pooled weighted effect size and 95% confidence interval, positive effect size: improved force sense accuracy for the no-taping group, negative effect size: improved force sense accuracy for the taping group.

**Figure S9**. Forest plot illustrating the effect of taping on relative force sense accuracy (healthy individuals). Black boxes: individual weighted effect sizes, whiskers: 95% confidence intervals, red diamond: pooled weighted effect size and 95% confidence interval, positive effect size: improved force sense accuracy for the no-taping group, negative effect size: improved force sense accuracy for the taping group.

**Figure S10**. Forest plot illustrating the effect of taping on relative force sense accuracy (individuals with medial epicondylitis). Black boxes: individual weighted effect sizes, whiskers: 95% confidence intervals, red diamond: pooled weighted effect size and 95% confidence interval, positive effect size: improved force sense accuracy for the no-taping group, negative effect size: improved force sense accuracy for the taping group.

**Figure S11**. Forest plot illustrating the effect of taping on absolute force sense accuracy. Black boxes: individual weighted effect sizes, whiskers: 95% confidence intervals, red diamond: pooled weighted effect size and 95% confidence interval, positive effect size: improved force sense accuracy for the placebo taping group, negative effect size: improved force sense accuracy for the taping group.

**Figure S12**. Forest plot illustrating the effect of taping on absolute force sense accuracy (healthy individuals). Black boxes: individual weighted effect sizes, whiskers: 95% confidence intervals, red diamond: pooled weighted effect size and 95% confidence interval, positive effect size: improved force sense accuracy for the placebo taping group, negative effect size: improved force sense accuracy for the taping group.

**Figure S13**. Forest plot illustrating the effect of taping on absolute force sense accuracy (individuals with medial epicondylitis). Black boxes: individual weighted effect sizes, whiskers: 95% confidence intervals, red diamond: pooled weighted effect size and 95% confidence interval, positive effect size: improved force sense accuracy for the placebo taping group, negative effect size: improved force sense accuracy for the taping group.

**Figure S14**. Forest plot illustrating the effect of taping on relative force sense accuracy. Black boxes: individual weighted effect sizes, whiskers: 95% confidence intervals, red diamond: pooled weighted effect size and 95% confidence interval, positive effect size: improved force sense accuracy for the placebo taping group, negative effect size: improved force sense accuracy for the taping group.

**Figure S15**. Forest plot illustrating the effect of taping on relative force sense accuracy (healthy individuals). Black boxes: individual weighted effect sizes, whiskers: 95% confidence intervals, red diamond: pooled weighted effect size and 95% confidence interval, positive effect size: improved force sense accuracy for the placebo taping group, negative effect size: improved force sense accuracy for the taping group.

**Figure S16**. Forest plot illustrating the effect of taping on relative force sense accuracy (individuals with medial epicondylitis). Black boxes: individual weighted effect sizes, whiskers: 95% confidence intervals, red diamond: pooled weighted effect size and 95% confidence interval, positive effect size: improved force sense accuracy for the placebo taping group, negative effect size: improved force sense accuracy for the taping group.

**Figure S17**. Forest plot illustrating the effect of taping on absolute force sense accuracy (repeated measures design). Black boxes: individual weighted effect sizes, whiskers: 95% confidence intervals, red diamond: pooled weighted effect size and 95% confidence interval, positive effect size: deterioration in force sense accuracy, negative effect size: improvement in force sense accuracy.

**Figure S18**. Forest plot illustrating the effect of taping on absolute force sense accuracy (elastic tape). Black boxes: individual weighted effect sizes, whiskers: 95% confidence intervals, red diamond: pooled weighted effect size and 95% confidence interval, positive effect size: deterioration in force sense accuracy, negative effect size: improvement in force sense accuracy.

**Figure S19**. Leave-one-out sensitivity analysis for between group analysis for absolute force sense accuracy (taping vs. no comparator). Black boxes: individual weighted effect sizes, whiskers: 95% confidence intervals, red diamond: pooled weighted effect size and 95% confidence interval, positive effect size: improvement in force sense accuracy for the no-taping group, negative effect size: improvement in force sense accuracy for the taping group

**Figure S20**. Leave-one-out sensitivity analysis for between group analysis for absolute force sense accuracy (repeated measures design, taping vs. no comparator). Black boxes: individual weighted effect sizes, whiskers: 95% confidence intervals, red diamond: pooled weighted effect size and 95% confidence interval, positive effect size: improvement in force sense accuracy for the no-taping group, negative effect size: improvement in force sense accuracy for the taping group

**Figure S21**. Leave-one-out sensitivity analysis for between group analysis for absolute force sense accuracy (elastic tape, elastic taping vs. no comparator). Black boxes: individual weighted effect sizes, whiskers: 95% confidence intervals, red diamond: pooled weighted effect size and 95% confidence interval, positive effect size: improvement in force sense accuracy for the no-taping group, negative effect size: improvement in force sense accuracy for the elastic taping group

**Figure S22**. Leave-one-out sensitivity analysis for between group analysis for absolute force sense accuracy (rigid tape, rigid taping vs. no comparator). Black boxes: individual weighted effect sizes, whiskers: 95% confidence intervals, red diamond: pooled weighted effect size and 95% confidence interval, positive effect size: improvement in force sense accuracy for the no-taping group, negative effect size: improvement in force sense accuracy for the rigid taping group

**Figure S23**. Leave-one-out sensitivity analysis for between group analysis for absolute force sense accuracy (healthy individuals, taping vs. no comparator). Black boxes: individual weighted effect sizes, whiskers: 95% confidence intervals, red diamond: pooled weighted effect size and 95% confidence interval, positive effect size: improvement in force sense accuracy for the no-taping group, negative effect size: improvement in force sense accuracy for the taping group

**Figure S24**. Leave-one-out sensitivity analysis for between group analysis for absolute force sense accuracy (individuals with medial epicondylitis, taping vs. no comparator). Black boxes: individual weighted effect sizes, whiskers: 95% confidence intervals, red diamond: pooled weighted effect size and 95% confidence interval, positive effect size: improvement in force sense accuracy for the no-taping group, negative effect size: improvement in force sense accuracy for the taping group

**Figure S25**. Leave-one-out sensitivity analysis for between group analysis for absolute force sense accuracy (healthy individuals, elastic taping vs. no comparator). Black boxes: individual weighted effect sizes, whiskers: 95% confidence intervals, red diamond: pooled weighted effect size and 95% confidence interval, positive effect size: improvement in force sense accuracy for the no-taping group, negative effect size: improvement in force sense accuracy for the elastic taping group

**Figure S26**. Leave-one-out sensitivity analysis for between group analysis for absolute force sense accuracy (healthy individuals, rigid taping vs. no comparator). Black boxes: individual weighted effect sizes, whiskers: 95% confidence intervals, red diamond: pooled weighted effect size and 95% confidence interval, positive effect size: improvement in force sense accuracy for the no-taping group, negative effect size: improvement in force sense accuracy for the rigid taping group

**Figure S27**. Leave-one-out sensitivity analysis for between group analysis for relative force sense accuracy (taping vs. no comparator). Black boxes: individual weighted effect sizes, whiskers: 95% confidence intervals, red diamond: pooled weighted effect size and 95% confidence interval, positive effect size: improvement in force sense accuracy for the no-taping group, negative effect size: improvement in force sense accuracy for the taping group

**Figure S28**. Leave-one-out sensitivity analysis for between group analysis for relative force sense accuracy (repeated measures design, taping vs. no comparator). Black boxes: individual weighted effect sizes, whiskers: 95% confidence intervals, red diamond: pooled weighted effect size and 95% confidence interval, positive effect size: improvement in force sense accuracy for the no-taping group, negative effect size: improvement in force sense accuracy for the taping group

**Figure S29**. Leave-one-out sensitivity analysis for between group analysis for relative force sense accuracy (healthy individuals, taping vs. no comparator). Black boxes: individual weighted effect sizes, whiskers: 95% confidence intervals, red diamond: pooled weighted effect size and 95% confidence interval, positive effect size: improvement in force sense accuracy for the no-taping group, negative effect size: improvement in force sense accuracy for the taping group

**Figure S30**. Leave-one-out sensitivity analysis for between group analysis for relative force sense accuracy (individuals with medial epicondylitis, taping vs. no comparator). Black boxes: individual weighted effect sizes, whiskers: 95% confidence intervals, red diamond: pooled weighted effect size and 95% confidence interval, positive effect size: improvement in force sense accuracy for the no-taping group, negative effect size: improvement in force sense accuracy for the taping group

**Figure S31**. Leave-one-out sensitivity analysis for between group analysis for absolute force sense accuracy (taping vs. placebo taping comparator). Black boxes: individual weighted effect sizes, whiskers: 95% confidence intervals, red diamond: pooled weighted effect size and 95% confidence interval, positive effect size: improvement in force sense accuracy for the placebo taping group, negative effect size: improvement in force sense accuracy for the taping group

**Figure S32**. Leave-one-out sensitivity analysis for between group analysis for absolute force sense accuracy (healthy individuals, taping vs. placebo taping comparator). Black boxes: individual weighted effect sizes, whiskers: 95% confidence intervals, red diamond: pooled weighted effect size and 95% confidence interval, positive effect size: improvement in force sense accuracy for the placebo taping group, negative effect size: improvement in force sense accuracy for the taping group

**Figure S33**. Leave-one-out sensitivity analysis for between group analysis for absolute force sense accuracy (individuals with medial epicondylitis, taping vs. placebo taping comparator). Black boxes: individual weighted effect sizes, whiskers: 95% confidence intervals, red diamond: pooled weighted effect size and 95% confidence interval, positive effect size: improvement in force sense accuracy for the placebo taping group, negative effect size: improvement in force sense accuracy for the taping group

**Figure S34**. Leave-one-out sensitivity analysis for between group analysis for relative force sense accuracy (taping vs. placebo taping comparator). Black boxes: individual weighted effect sizes, whiskers: 95% confidence intervals, red diamond: pooled weighted effect size and 95% confidence interval, positive effect size: improvement in force sense accuracy for the placebo taping group, negative effect size: improvement in force sense accuracy for the taping group

**Figure S35**. Leave-one-out sensitivity analysis for between group analysis for relative force sense accuracy (healthy individuals, taping vs. placebo taping comparator). Black boxes: individual weighted effect sizes, whiskers: 95% confidence intervals, red diamond: pooled weighted effect size and 95% confidence interval, positive effect size: improvement in force sense accuracy for the placebo taping group, negative effect size: improvement in force sense accuracy for the taping group

**Figure S36**. Leave-one-out sensitivity analysis for between group analysis for relative force sense accuracy (individuals with medial epicondylitis, taping vs. placebo taping comparator). Black boxes: individual weighted effect sizes, whiskers: 95% confidence intervals, red diamond: pooled weighted effect size and 95% confidence interval, positive effect size: improvement in force sense accuracy for the placebo taping group, negative effect size: improvement in force sense accuracy for the taping group

**Figure S37**. Leave-one-out sensitivity analysis for within group analysis for absolute force sense accuracy. Black boxes: individual weighted effect sizes, whiskers: 95% confidence intervals, red diamond: pooled weighted effect size and 95% confidence interval, positive effect size: deterioration in force sense accuracy, negative effect size: improvement in force sense accuracy

**Figure S38**. Leave-one-out sensitivity analysis for within group analysis for absolute force sense accuracy (repeated measures design). Black boxes: individual weighted effect sizes, whiskers: 95% confidence intervals, red diamond: pooled weighted effect size and 95% confidence interval, positive effect size: deterioration in force sense accuracy, negative effect size: improvement in force sense accuracy

**Figure S39**. Leave-one-out sensitivity analysis for within group analysis for absolute force sense accuracy (elastic tape). Black boxes: individual weighted effect sizes, whiskers: 95% confidence intervals, red diamond: pooled weighted effect size and 95% confidence interval, positive effect size: deterioration in force sense accuracy, negative effect size: improvement in force sense accuracy.

**Search strategy for individual databases**

**Web of Science**

TS=((Healthy* or Neurological disorders* or neurological disease* or neuropathy* or musculoskeletal disorder* or musculoskeletal disease* or myopathy*) AND (Age groups* or adolescent* or young* or elderly* or old*) AND (athlete* or elite athlete* or recreational athlete* or novice athlete* or trained athlete* or sedentary*) AND (Tape* or taping* or orthotic tape* or kinesiotape* or kinesio tape* or leukotape* or leuko tape* or rock tape* or spiral tape* or spider tape* or mcconnell tape* or rigid tape* or athletic tape* or elastic tape* or patellar tape* or adhesive tape* or gidney tape*) AND, OR (control group* or control* or placebo* or placebo group* or sham* or sham group*) AND, OR (joint* or shoulder* or knee* or hip* or ankle* or elbow* or wrist* or spine* or neck* or lumbar* or thoracic* or cervical*) AND (force sense error* or force sense error test* or force sense accuracy* or fset* or fse* or sense of force* or force sense measurement* or force sense* or sense of force measurement* or sense of force* or force sensing* or maximum voluntary isometric contraction reproduction* or mvic reproduction*) AND (clinical trial* or intervention study* or cohort analysis* or longitudinal study* or cluster analysis* or crossover trial* or cluster analysis* or randomized trial* or major clinical study*))

**Pubmed**

(Healthy or Neurological disorders or neurological disease or neuropathy or musculoskeletal disorder or musculoskeletal disease or myopathy) AND (Age groups or adolescent or young or elderly or old) AND (athlete or elite athlete or recreational athlete or novice athlete or trained athlete or sedentary) AND (Tape or taping or orthotic tape or kinesiotape or kinesio tape or leukotape or leuko tape or rock tape or spiral tape or spider tape or mcconnell tape or rigid tape or athletic tape or elastic tape or patellar tape or adhesive tape or gidney tape) AND, OR (control group or control or placebo or placebo group or sham or sham group) AND, OR (joint or shoulder or knee or hip or ankle or elbow or wrist or spine or neck or lumbar or thoracic or cervical) AND (force sense error or force sense error test or force sense accuracy or fset or fse or sense of force or force sense measurement or force sense or sense of force measurement or sense of force or force sensing or maximum voluntary isometric contraction reproduction or mvic reproduction) AND (clinical trial or intervention study or cohort analysis or longitudinal study or cluster analysis or crossover trial or cluster analysis or randomized trial or major clinical study)

**CENTRAL**

#1 (Healthy or Neurological disorders or neurological disease or neuropathy or musculoskeletal disorder or musculoskeletal disease or myopathy AND Age groups or adolescent or young or elderly or old AND athlete or elite athlete or recreational athlete or novice athlete or trained athlete or sedentary):ti,ab,kw

#2 (Tape or taping or orthotic tape or kinesiotape or kinesio tape or leukotape or leuko tape or rock tape or spiral tape or spider tape or mcconnell tape or rigid tape or athletic tape or elastic tape or patellar tape or adhesive tape or gidney tape):ti,ab,kw

#3 (control group or control or placebo or placebo group or sham or sham group OR joint or shoulder or knee or hip or ankle or elbow or wrist or spine or neck or lumbar or thoracic or cervical):ti,ab,kw

#4 (force sense error or force sense error test or force sense accuracy or fset or fse or sense of force or force sense measurement or force sense or sense of force measurement or sense of force or force sensing or maximum voluntary isometric contraction reproduction or mvic reproduction):ti,ab,kw

#5 (clinical trial or intervention study or cohort analysis or longitudinal study or cluster analysis or crossover trial or cluster analysis or randomized trial or major clinical study):ti,ab,kw

#12 1 and 2 and 3 and 4 and 5

**EMBASE**

#1 (‘healthy’ OR ‘neurological disorders’ OR ‘neurological disease’ OR ‘neuropathy’ OR ‘musculoskeletal disorder’ OR ‘musculoskeletal disease’ OR ‘myopathy’)/de OR (healthy OR neurological disorders OR neurological disease OR neuropathy OR musculoskeletal disorder OR musculoskeletal disease OR myopathy);ti;ab

#2 ((‘age groups’ OR ‘adolescent’ OR ‘young’ OR ‘elderly’ OR ‘old’) OR (‘athlete’ OR ‘elite athlete’ OR ‘recreational athlete’ OR ‘novice athlete’ OR ‘trained athlete’ OR ‘sedentary’))/de OR ((age groups OR adolescent OR young OR elderly OR old OR (athlete OR elite athlete OR recreational athlete OR novice athlete OR trained athlete OR sedentary));ti;ab

#3 (‘Tape’ OR ‘taping’ OR ‘orthotic tape’ OR ‘kinesiotape’ OR ‘kinesio tape’ OR ‘leukotape’ OR ‘leuko tape’ OR ‘rock tape’ OR ‘spiral tape’ OR ‘spider tape’ OR ‘mcconell tape’ OR ‘rigid tape’ OR ‘athletic tape’ OR ‘elastic tape’ OR ‘patellar tape’ OR ‘adhesive tape’ OR ‘gibney tape’)/de OR (Tape OR taping OR orthotic tape OR kinesiotape OR kinesio tape OR leukotape OR leuko tape OR rock tape OR spiral tape OR spider tape OR McConnell tape OR rigid tape OR athletic tape OR elastic tape OR patellar tape OR adhesive tape OR gibney tape);ti,ab

#4 (‘control group’ OR ‘control’ OR ‘placebo’ OR ‘placebo group’ OR ‘sham’ or ‘sham group’)/de OR (control group OR control OR placebo OR placebo group OR sham or sham group);ti;ab

#5 (‘joint’ OR ‘shoulder’ OR ‘knee’ OR ‘hip’ OR ‘ankle’ OR ‘elbow’ OR ‘wrist’ OR ‘spine’ OR ‘neck’ or ‘lumbar’ OR ‘thoracic’ OR ‘cervical’)/de OR (joint OR shoulder OR knee OR hip OR ankle OR elbow OR wrist OR spine OR neck or lumbar OR thoracic OR cervical):ti,ab

#6 (‘force sense error’ OR ‘force sense error test’ OR ‘FSET’ OR ‘FSE’ OR ‘sense of force’ OR ‘force sense measurement’ OR ‘force sense’ OR ‘sense of force measurement’ OR ‘sense of force’ OR ‘force sensing’ OR ‘maximum voluntary isometric contraction reproduction’ OR ‘MVIC reproduction’)/de OR (force sense error OR force sense error test OR FSET OR FSE OR sense of force OR force sense measurement OR force sense OR sense of force measurement OR sense of force OR force sensing OR maximum voluntary isometric contraction reproduction OR MVIC reproduction);ti,ab

#7 #1 and #2 and #3 and, or #4 and, or #5 and #6

**PsychInfo**

(Healthy OR Neurological disorders OR neurological disease OR neuropathy OR musculoskeletal disorder OR musculoskeletal disease OR myopathy) AND (Age groups OR adolescent OR young OR elderly OR old) AND (athlete OR elite athlete OR recreational athlete OR novice athlete OR trained athlete OR sedentary) AND (Tape OR taping OR orthotic tape OR kinesiotape OR kinesio tape OR leukotape OR leuko tape OR rock tape OR spiral tape OR spider tape OR mcconnell tape OR rigid tape OR athletic tape OR elastic tape OR patellar tape OR adhesive tape OR gidney tape) AND (control group OR control OR placebo OR placebo group OR sham OR sham group) OR (joint OR shoulder OR knee OR hip OR ankle OR elbow OR wrist OR spine OR neck OR lumbar OR thoracic OR cervical) AND (force sense error OR force sense error test OR force sense accuracy OR fset OR fse OR sense of force OR force sense measurement OR force sense OR sense of force measurement OR sense of force OR force sensing OR maximum voluntary isometric contraction reproduction OR mvic reproduction) AND (clinical trial OR intervention study OR cohort analysis OR longitudinal study OR cluster analysis OR crossover trial OR cluster analysis OR randomized trial OR major clinical study)

**EBSCO host**

(Healthy OR Neurological disorders OR neurological disease OR neuropathy OR musculoskeletal disorder OR musculoskeletal disease OR myopathy) AND (Age groups OR adolescent OR young OR elderly OR old) AND (athlete OR elite athlete OR recreational athlete OR novice athlete OR trained athlete OR sedentary) AND (Tape OR taping OR orthotic tape OR kinesiotape OR kinesio tape OR leukotape OR leuko tape OR rock tape OR spiral tape OR spider tape OR mcconnell tape OR rigid tape OR athletic tape OR elastic tape OR patellar tape OR adhesive tape OR gidney tape) OR (control group OR control OR placebo OR placebo group OR sham OR sham group) OR (joint OR shoulder OR knee OR hip OR ankle OR elbow OR wrist OR spine OR neck OR lumbar OR thoracic OR cervical) AND (force sense error OR force sense error test OR force sense accuracy OR fset OR fse OR sense of force OR force sense measurement OR force sense OR sense of force measurement OR sense of force OR force sensing OR maximum voluntary isometric contraction reproduction OR mvic reproduction) AND (clinical trial OR intervention study OR cohort analysis OR longitudinal study OR cluster analysis OR crossover trial OR cluster analysis OR randomized trial OR major clinical study)

**PEDro***

1. Force sense error
2. Force sense accuracy
3. Force sense
4. Force sense tape
5. Force sense taping

*Search performed individually for each term

**Scopus**

( ALL ( healthy OR neurological AND disorders OR neurological AND disease OR neuropathy OR musculoskeletal AND disorder OR musculoskeletal AND disease OR myopathy ) AND ALL ( age AND groups OR adolescent OR young OR elderly OR old ) AND ALL ( athlete OR elite AND athlete OR recreational AND athlete OR novice AND athlete OR trained AND athlete OR sedentar ) AND ALL ( tape OR taping OR orthotic AND tape OR kinesiotape OR kinesio AND tape OR leukotape OR leuko AND tape OR rock AND tape OR spiral AND tape OR spider AND tape OR mcconnell AND tape OR rigid AND tape OR athletic AND tape OR elastic AND tape OR patellar AND tape OR adhesive AND tape OR gidney AND tape ) OR ALL ( control AND group OR control OR placebo OR placebo AND group OR sham OR sham AND group ) OR ALL ( joint OR shoulder OR knee OR hip OR ankle OR elbow OR wrist OR spine OR neck OR lumbar OR thoracic OR cervical ) AND ALL ( force AND sense AND error OR force AND sense AND error AND test OR force AND sense AND accuracy OR fset OR fse OR sense AND of AND force OR force AND sense AND measurement OR force AND sense OR sense AND of AND force AND measurement OR sense AND of AND force OR force AND sensing OR maximum AND voluntary AND isometric AND contraction AND reproduction OR mvic AND reproduction ) AND ALL ( clinical AND trial OR intervention AND study OR cohort AND analysis OR longitudinal AND study OR cluster AND analysis OR crossover AND trial OR cluster AND analysis OR randomized AND trial OR major AND clinical AND study ) )
